# Conversion of Spin‐Orbit‐Coupled Optical Vortices via Complex Coupling

**DOI:** 10.1002/nap2.70337

**Published:** 2026-10-23

**Authors:** Jinze Gao, Xin Xie, Kun Liao, Xiaoyong Hu

**Affiliations:** ^1^ State Key Laboratory for Mesoscopic Physics & Department of Physics Collaborative Innovation Center of Quantum Matter & Frontiers Science Center for Nano‐optoelectronics Peking University Beijing China; ^2^ Key Laboratory for Advanced Optoelectronic Integrated Chips of Jiangsu Province Peking University Yangtze Delta Institute of Optoelectronics Nantong Jiangsu China; ^3^ Collaborative Innovation Center of Extreme Optics Shanxi University Taiyuan Shanxi China; ^4^ Hefei National Laboratory Hefei China

**Keywords:** complex coupling, dynamic angular momentum conversion, floquet photonics, optical vortices, orbital angular momentum, spin‐orbit interaction

## Abstract

Optical vortices provide an additional freedom because of the orbital angular momentum (OAM), enabling significant advances in high‐dimensional information encoding, quantum communication, and super‐resolution imaging. However, the controlled conversion between spin‐orbit‐coupled (SOC) optical vortices of different orders remains challenging. Here, we propose a novel four‐step Floquet driving protocol governed by complex coupling to realize conversion between distinct angular momentum characteristics in strongly confined waveguide arrays. Optical vortices naturally give rise to complex‐valued coupling coefficients owing to their intrinsic spatial‐phase structure and spin–orbit interaction. Building on this mechanism, we implement a Floquet driving protocol in the waveguide array, which supports corner and edge states determined by the coupling parameters. Exploiting the corner states, we demonstrate dynamic conversion of total‐angular‐momentum order from j to −j across successive two or three driving periods. This work opens a pathway toward dynamic modulation of structured light and advances the integration of OAM‐based functionalities in photonic platforms.

## Graphical abstract

A four‐step Floquet protocol in a straight terahertz waveguide array uses metallic separators to selectively control interwaveguide coupling. The resulting angular‐momentum‐dependent coupling enables Floquet engineering of OAM states, giving rise to corner states, conversion between opposite total‐angular‐momentum states, and edge states.

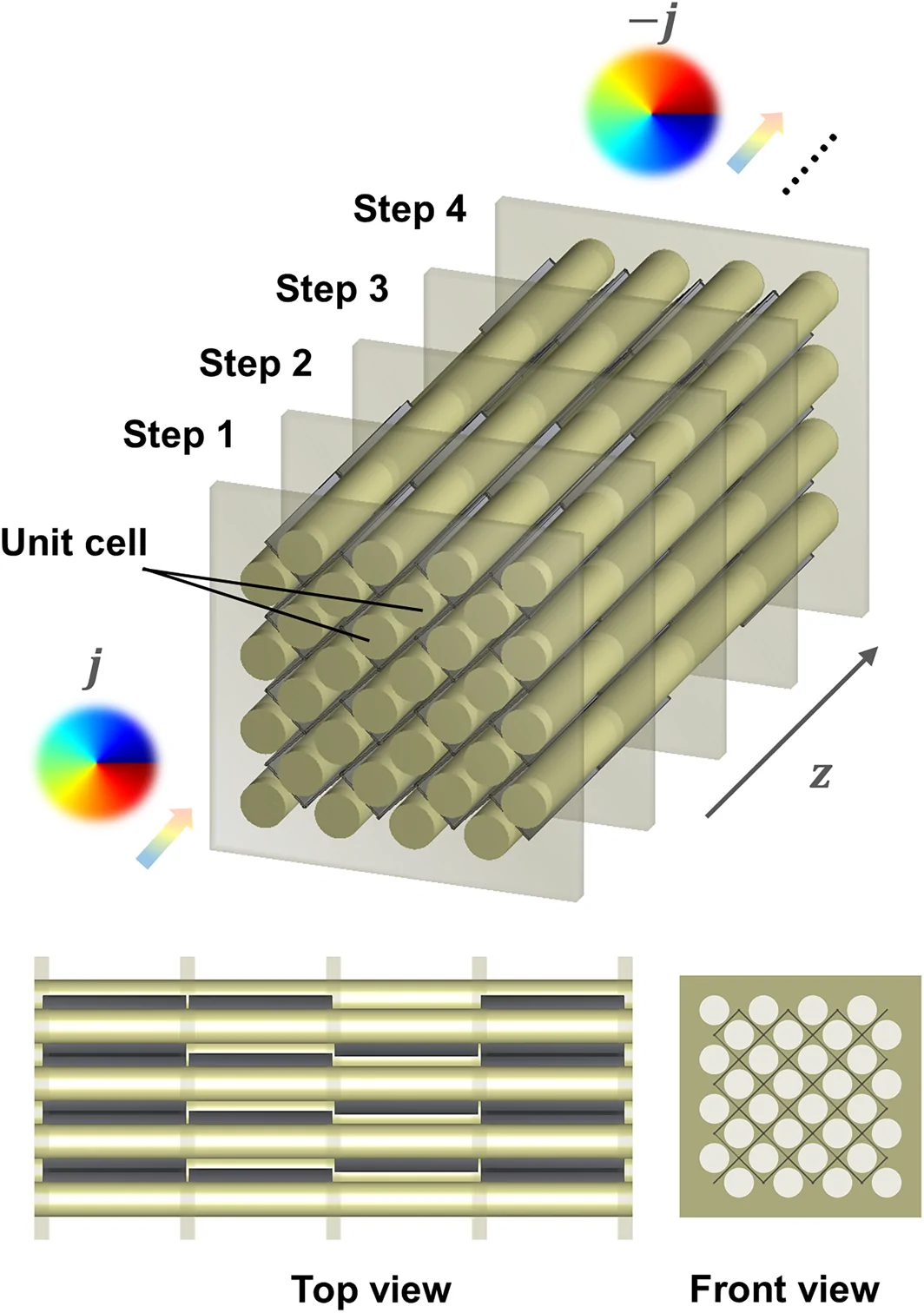

## 1 Introduction

Optical vortices with OAM provide a unique degree of freedom for modulating light, first proposed in 1992 [1]. Since then, it has been widely studied and applied in areas such as high‐dimensional information encoding [2, 3], quantum communication [4], super‐resolution imaging [5, 6, 7], particle manipulation [8], and integrated photonics [9, 10, 11, 12, 13]. In recent years, progress in diffractive optics, spatial light modulators, metasurfaces, and integrated photonic technologies [14, 15, 16, 17] has greatly improved the generation, modulation, and detection of optical vortices [18, 19, 20, 21]. Among various approaches, ring resonators, helical‐phase gratings, and electro‐optic modulators are commonly used in on‐chip OAM devices [22, 23, 24, 25, 26, 27]. These technologies are key to enabling high‐density OAM‐multiplexed optical communication systems [28]. However, efficient conversion between optical vortices in integrated photonic structures remains highly challenging.

In parallel, cyclic driving protocols in photonic systems have emerged as a powerful framework for dynamic control through the introduction of time‐periodic Hamiltonians. A wide range of physical phenomena have been demonstrated, including flat‐band generation [29], Bloch oscillations [30], subharmonic edge‐state dynamics at zero and π quasi‐energies [31], and higher‐order corner‐state oscillations [32]. Yet most of these studies have concentrated on fundamental modes. The integration of optical vortices with cyclic driving protocols remains largely unexplored, despite its potential to enable dynamic control over optical vortices and to open new pathways for manipulating structured light.

In this work, we combine optical vortices with four‐step Floquet driving in strongly confined circular waveguide arrays, where the refractive‐index contrast between the waveguides and the surrounding medium is large, and the waveguide diameters are comparable to the optical wavelength. Owing to their intrinsic helical phase structure, OAM modes naturally introduce complex phase terms into interwaveguide coupling coefficients, as previously reported [13]. Although complex coupling has been widely studied before, a key limitation arises from the modal properties of light, which typically restrict the tunable range of the complex phase [13, 29, 33, 34, 35]. In our system, the eigenmodes of strongly confined waveguides exhibit pronounced spin–orbit coupling (SOC), accompanied by substantial longitudinal field components and a fully vectorial field structure. The transverse circular‐basis components are characterized by the index σ=±1, with their azimuthal indices satisfying l=j−σ, where j denotes the axial total‐angular‐momentum index of the full‐vector mode. The phase of the complex‐valued coupling coefficient is determined by the azimuthal phase structure of the coupled field components and therefore depends on their OAM indices l. Consequently, different spin–orbit branches exhibit distinct coupling characteristics. Furthermore, we implement a cyclic driving protocol in the strongly confined waveguide array supporting optical vortices. The protocol consists of four distinct steps with different coupling configurations, such that a given waveguide sequentially couples to four nearest neighbors. We first consider the case in which only optical‐vortex modes with the same total‐angular‐momentum index j are coupled, leading to corner states in the quasi‐energy spectrum. By modulating the coupling coefficients, the total‐angular‐momentum index of the corner state reverses from j to −j. When coupling between modes with different j is allowed, the system instead supports edge states, leading to optical‐field localization along the boundaries of the waveguide array. These findings demonstrate that waveguide arrays supporting optical vortices provide a versatile platform for realizing unconventional structured‐light dynamics.

We compare our work with existing photonic platforms employing different mechanisms for engineering complex in following Table 1.

**TABLE 1:** Comparison of this work with existing photonic platforms employing different mechanisms for engineering complex coupling.

	Platform	Principles of coupling	Phase range	Wavelength	Physical phenomena
[33]	Waveguide array	Periodically curve the waveguide	0 or π	1550 nm (193.4 THz)	Artificial gauge field
[28]	Micro‐ring resonator lattice	Shift the auxiliary rings to introduce a magnetic flux	0 or π	/	Flat‐belt photon control
[34]	Waveguide array	Introduce loss via metal‐containing auxiliary waveguides	[−π/2,0]	1550 nm (193.4 THz)	APT‐symmetry, Anti‐hermitian system
[35]	Waveguide array	Under weak confinement, generate {0,π} phase through the characteristics of the d mode.	0 or π	1550 nm (193.4 THz)	Mobius topological insulator
[13]	Waveguide array	Under weak confinement, generate complex terms due to OAM	[0,2π)	1550 nm (193.4 THz) [6], 532 nm (563.5 THz) [7]	Controllable artificial guage fields [6], Stable OAM transmission [7]
This work	Waveguide array	Under strong confinement, generate complex terms naturally due to both OAM and SAM	[0,2π)	750 μm (0.4 THz)	Corner and edge state, Conversion between OVs of different OAM and SAM orders

## 2 Results and Discussion

### 2.1 Complex Coupling Between Optical Vortices of Identical Order

In weakly confined systems, polarization has minimal influence on mode coupling. In contrast, in strongly confined waveguides, where spin–orbit interaction becomes prominent, the coupling exhibits a pronounced dependence on the spin angular momentum of the field [36, 37]. In this work, we consider a strongly confined system operating in the terahertz frequency range, where circular waveguides are fabricated using 3D‐printed resin materials. By solving Maxwell's equations for an infinitely long circular waveguide, we obtain the electric‐field eigenmodes for a waveguide with core refractive index $n_{1}$, background refractive index $n_{0}$, and radius r. Let the basis vectors of the Cartesian coordinate system be $\left\{e_{x},e_{y},e_{z}\right\}$. The transverse electric field can be expressed in the circular basis as $e_{\sigma }$, with σ=±1. The complete electric‐field eigenmode can then be written as a superposition of the two transverse circular‐basis components together with the longitudinal component (Supporting Information S1). For a full‐vector eigenmode with axial rotational eigenvalue j=n, defined through the following equation [38]:

(1) $$\begin{array}{c} \hat{J_{z}}E=jE,\hat{J_{z}}=\hat{L_{z}}+\hat{S_{z}}, \end{array}$$

The transverse circular‐basis components satisfy l=j−σ. Accordingly, the σ=−1 component has l=n+1, whereas the σ=+1 component has l=n−1. The longitudinal field carries an azimuthal dependence $E_{z}\propto e^{in\varphi }$, corresponding to $l_{z}=n$. Here, σ=±1 denotes the circular‐spin basis index of the transverse field and should not be identified with the actual spin angular momentum per photon of the strongly confined mode. Because of the nonnegligible longitudinal fields, the local spin density is generally inhomogeneous, and the cross‐section‐averaged modal spin angular momentum is not restricted to ±ℏ. Nevertheless, the complete vector mode remains characterized by the definite rotational eigenvalue j=n.

Using the same approach, the magnetic field can also be divided into two parts (Supporting Information S1). Analysis of the eigenvalue equation, together with the numerically obtained waveguide modes, shows that for a given rotational eigenvalue j=n, two distinct modal branches exist, as illustrated in Figure 1a. These modes have different effective refractive indices and are dominated by opposite transverse circular‐basis components, although both belong to the same rotational sector j=n.

**FIGURE 1:**
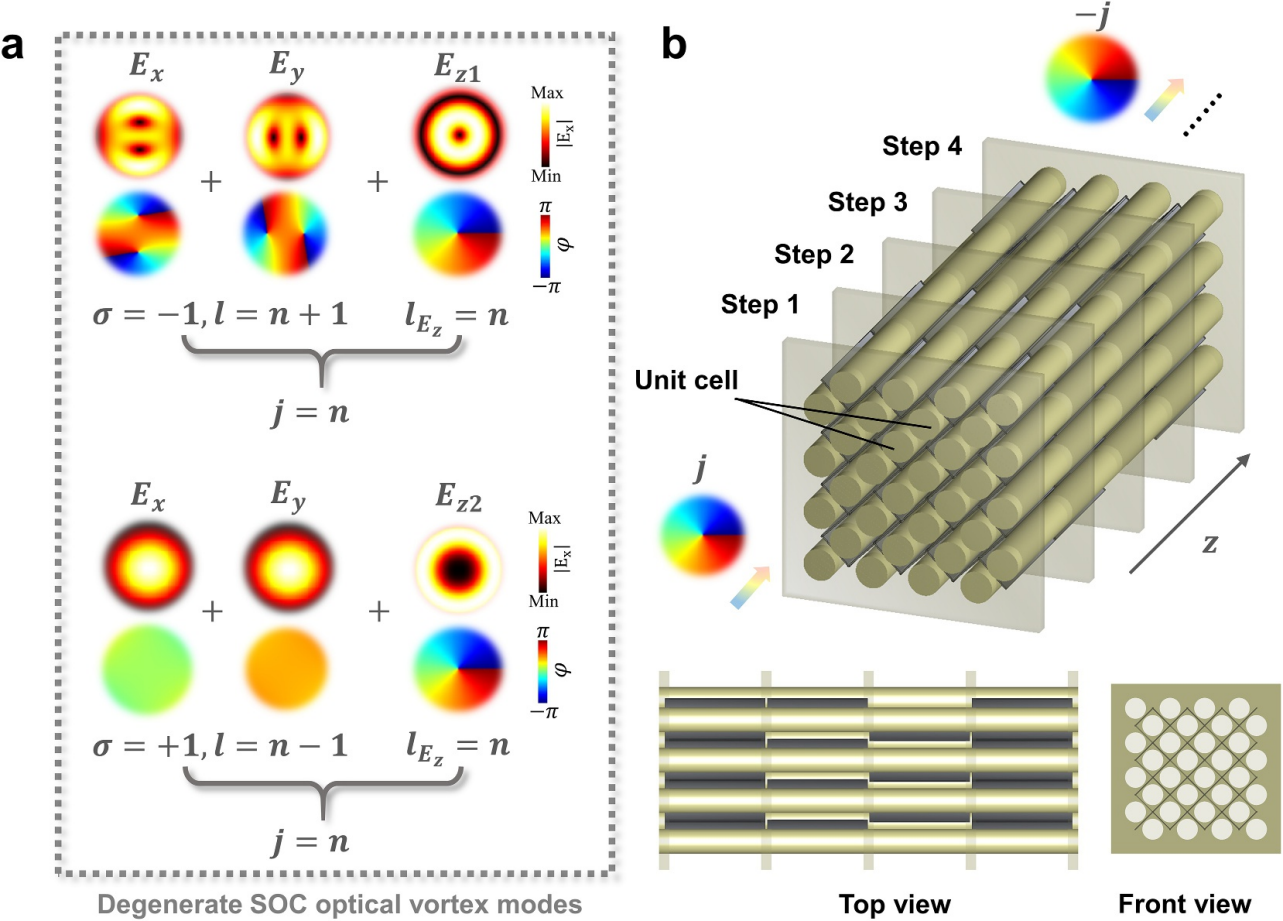
Four‐step Floquet driving protocol based on waveguide arrays supporting optical‐vortex modes. (a) Full‐vector spin–orbit‐coupled eigenmodes in a strongly confined waveguide. For a rotational eigenvalue j=n, the transverse circular‐basis components satisfy l=n−σ with σ=±1, whereas the longitudinal component has $l_{z}=n$. Here, σ denotes the circular‐spin basis index rather than the actual modal spin angular momentum. (b) Schematic of the four‐step Floquet driving protocol.

The coupled‐mode equations can then be solved analytically. The solution is as follows:

(2) $$\begin{array}{c} A_{n}\left(z\right)=\sum_{s}A_{s,n}e^{-i\kappa_{s}z}, \end{array}$$

where $\left\{\kappa_{s},A_{s,\text{an}}\right\}$ are the eigenvalues and eigenvectors of κ(dβ). Consider the coupling between modes with total angular momentum j and −j. It can be shown that $\kappa_{14}=\frac{-\omega \epsilon_{0}}{S}\iint_{S_{a}}\left(n_{1}^{2}-n_{0}^{2}\right)E_{1}^{*}\left(x,y\right)\cdot E_{4}\left(x,y\right)dxdy==\kappa_{0}{*e}^{-2i\theta_{z}}*e^{-i*2\left(l+1\right)\theta }$. Calculations for other coupling cases are provided in Supporting Information S1.

We consider two waveguides of identical radii in air, with an azimuthal angle $\theta_{12}$ and a separation distance D as presented in Figure 2a. Assuming l>0, the relevant set of modes is $\left\{E_{l,1},E_{-l,-1},E_{l,1},E_{-l,-1}\right\}\to \left\{E_{1},E_{2},E_{3},E_{4}\right\}$. The coupling matrix κ is given by the following equation:

(3) $$\begin{array}{c} \kappa =\left(\begin{array}{cccc} 0 & 0 & \kappa_{13}^{\left(0\right)} & \kappa_{14}^{\left(0\right)}e^{-i2\left(l+1\right)\theta_{12}}\\ 0 & 0 & {\kappa_{14}^{\left(0\right)}}^{*}e^{i2\left(l+1\right)\theta_{12}} & {\kappa_{13}^{\left(0\right)}}^{*}\\ {\kappa_{13}^{\left(0\right)}}^{*} & \kappa_{14}^{\left(0\right)}e^{-i2\left(l+1\right)\theta_{12}} & 0 & 0\\ {\kappa_{14}^{\left(0\right)}}^{*}e^{i2\left(l+1\right)\theta_{12}} & \kappa_{13}^{\left(0\right)} & 0 & 0 \end{array}\right). \end{array}$$

**FIGURE 2:**
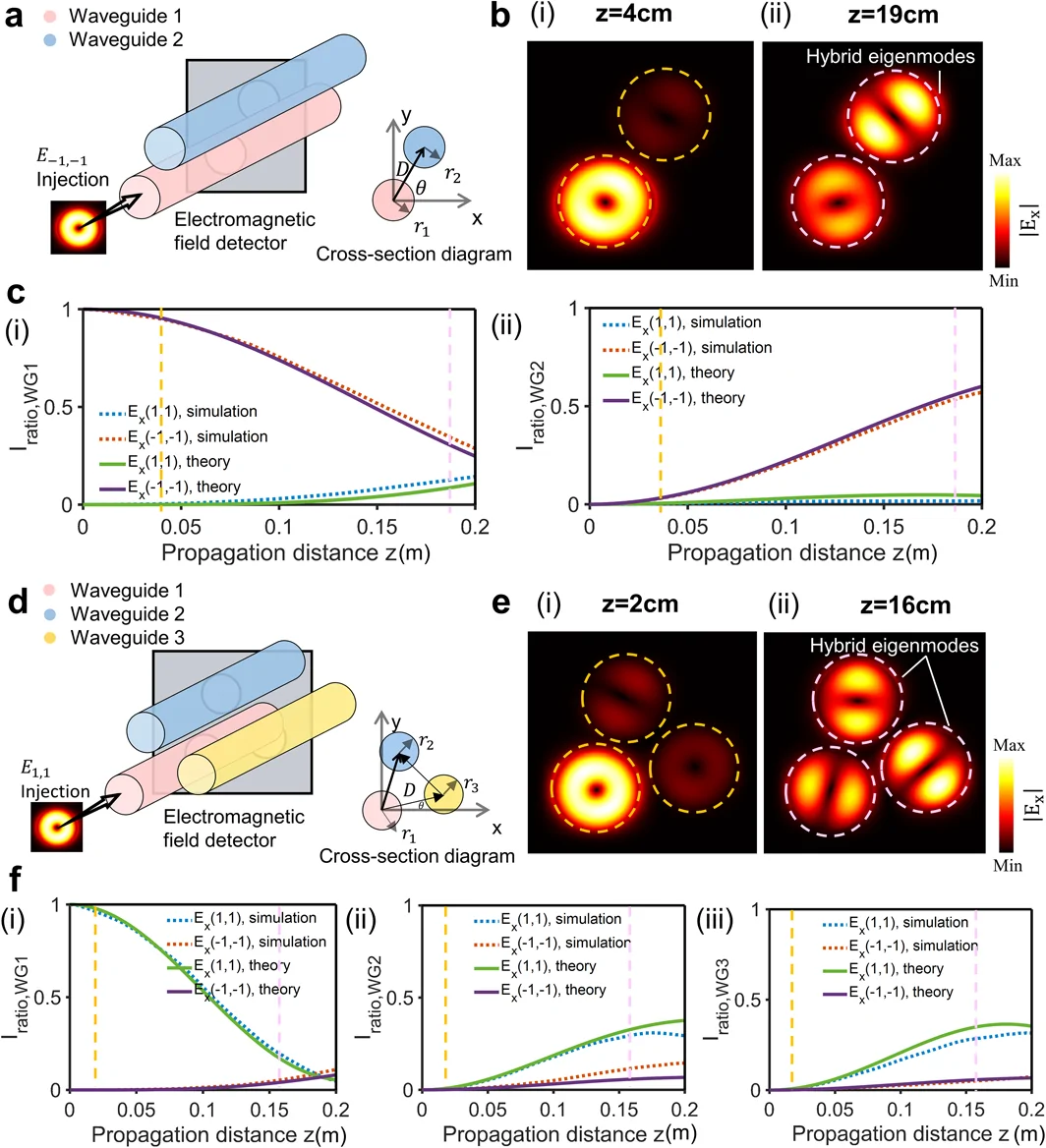
Two‐ and three‐waveguide structures and the propagation‐induced evolution of the eigenmode intensity distribution in each waveguide. (a) Schematic of the two‐waveguide structure. (b) Simulated electric field magnitude distribution (x‐polarized component) at propagation distances z=4cm and z=19cm. (c) Comparison of theoretical and simulated intensity ratios of eigenmodes during the state evolution in waveguide 1 (WG1) and waveguide 2 (WG2). (d) Schematic of the three‐waveguide structure. (e) Simulated electric field magnitude distribution (x‐polarized component) at propagation distances z=2cmandz=16cm. (f) Comparison of theoretical and simulated intensity ratios of eigenmodes during state evolution in WG1, 2 and 3.

If the waveguide slightly deviates from an ideal circular shape, then the two originally degenerate modes in the expression $E_{n}$ will become nearly degenerate, with effective propagation constants β,β+dβ. The correction due to near‐degeneracy is as follows:

(4) $$\begin{array}{c} \kappa \left(d\beta \right)=O^{-1}\left(O\kappa O^{-1}-\left(\begin{array}{cccc} 0 & 0 & 0 & 0\\ 0 & d\beta & 0 & 0\\ 0 & 0 & 0 & 0\\ 0 & 0 & 0 & d\beta \end{array}\right)\right)O=\kappa -O^{-1}\left(\begin{array}{cccc} 0 & 0 & 0 & 0\\ 0 & d\beta & 0 & 0\\ 0 & 0 & 0 & 0\\ 0 & 0 & 0 & d\beta \end{array}\right)O, \end{array}$$

where the transformation matrix O is defined as follows:

(5) $$\begin{array}{c} O=\left(\begin{array}{cccc} 1 & 1 & 0 & 0\\ i & -i & 0 & 0\\ 0 & 0 & 1 & 1\\ 0 & 0 & i & -i \end{array}\right). \end{array}$$

The waveguides are arranged with a refractive index $n_{1}=1.7$, and the surrounding spatial refractive index is $n_{0}=1.0$. The wavelength of the terahertz (THz) light in vacuum is λ=750μm. Both waveguides have a radius of $r_{1}=r_{2}=600\;\mu m$, and the center‐to‐center spacing between them is D=1400μm. The azimuthal angle between the two waveguides is $\theta =\frac{\pi }{3}$. The initial light is injected into waveguide 1, and the input mode is $E_{-1,-1}$. The corresponding mode set is denoted as $\left\{E_{1},E_{2},E_{3},E_{4}\right\}\to \left\{E_{1,1},E_{-1,-1},E_{2,1},E_{-2,-1}\right\}$. Because of the finite numerical discretization of the waveguides, the near‐degeneracy effect is taken into account for lower‐order modes in FDTD simulation. The propagation constant mismatch is given by $d\beta =k{\Delta n}_{\text{eff}}=-1.52,k=2\pi /\lambda$.

After propagating over a finite distance, the optical field initially confined in waveguide 1 couples into waveguide 2. The coupled field evolves into hybrid eigenmodes, as illustrated in Figure 2b. The optical field distribution at z = 4 cm corresponds to the position indicated by the yellow dashed line, whereas the field distribution at z = 16 cm corresponds to the position marked by the pink dashed line. It is shown that the theoretical predictions align well with the simulation results (Figure 2c). The simulated values of the coupling coefficients also match the theoretical predictions. Under near‐degenerate conditions, the central symmetry of the system with respect to the central axis is broken. This symmetry breaking enables the phase dependence of the coupling coefficients to manifest clearly in the dual‐waveguide system.

Three identical waveguides are considered in air, each with radius r, and arranged with azimuthal angles $\theta_{12},\theta_{13},\theta_{23}$, and respective center‐to‐center spacings $D_{1},D_{2}{,D}_{3}$ as presented in Figure 2d. Assuming l>0, the relevant set of modes is $\left\{E_{l,1},E_{-l,-1},E_{l,1},E_{-l,-1},E_{l,1},E_{-l,-1}\right\}\to \left\{E_{1},E_{2},E_{3},E_{4},E_{5},E_{6}\right\}$. Based on the previous discussions, the coupling constants in the multiwaveguide system can be obtained by extending the dual‐waveguide results. The coupling matrix κ is given by the following equation:

(6) $$\begin{array}{c} \kappa =\left(\begin{array}{cccccc} 0 & 0 & \kappa_{1} & \kappa_{2}e^{-i2\left(l+1\right)\theta_{12}} & \kappa_{3} & \kappa_{4}e^{-i2\left(l+1\right)\theta_{13}}\\ 0 & 0 & \kappa_{2}^{*}e^{i2\left(l+1\right)\theta_{12}} & \kappa_{1}^{*} & \kappa_{4}^{*}e^{i2\left(l+1\right)\theta_{13}} & \kappa_{3}^{*}\\ \kappa_{1}^{*} & \kappa_{2}e^{-i2\left(l+1\right)\theta_{12}} & 0 & 0 & \kappa_{5} & \kappa_{6}e^{-i2\left(l+1\right)\theta_{23}}\\ \kappa_{2}^{*}e^{i2\left(l+1\right)\theta_{12}} & \kappa_{1} & 0 & 0 & \kappa_{6}^{*}e^{i2\left(l+1\right)\theta_{23}} & \kappa_{5}^{*}\\ \kappa_{3}^{*} & \kappa_{4}e^{-i2n\left(l+1\right)} & \kappa_{5}^{*} & \kappa_{6}e^{-i2\left(l+1\right)\theta_{23}} & 0 & 0\\ \kappa_{4}^{*}e^{i2\left(l+1\right)\theta_{13}} & \kappa_{3} & \kappa_{6}^{*}e^{i2\left(l+1\right)\theta_{23}} & \kappa_{5} & 0 & 0 \end{array}\right). \end{array}$$

The waveguides are arranged in an equilateral triangle, each with a refractive index $n_{1}=1.7$, embedded in a background with refractive index $n_{0}=1.0$. The THz light has a vacuum wavelength λ=750μm. All waveguides have the same radius $r_{1}=r_{2}=r_{3}=600\;\mu m$, and the center‐to‐center spacing between them is D=1400μm. The azimuthal angle between waveguides 1 and 2 is $\theta_{12}=0.235$. The initial light is incident on waveguide 1 in the mode $E_{1,1}$, $\left\{E_{1},E_{2},E_{3},E_{4},E_{5},E_{6}\right\}\to \left\{E_{1,1},E_{-1,-1},E_{1,1},E_{-1,-1},E_{1,1},E_{-1,-1}\right\}$. Similar to the dual‐waveguide situation discussed previously, near‐degenerate effects must be considered for lower‐order modes, with $d\beta =kn_{\text{eff}}=-1.52,k=2\pi /\lambda$ (Supporting Information S1). In this symmetric triple‐waveguide system, the coupling coefficients satisfy $\kappa_{1}\left(\theta \right)=\kappa_{3}\left(\theta \right)=\kappa_{5}\left(\theta \right),\kappa_{2}\left(\theta \right)=\kappa_{4}\left(\theta \right)=\kappa_{6}\left(\theta \right)$. These correspond to $\kappa_{13}\left(\theta \right),\kappa_{14}\left(\theta \right)$ from the dual‐waveguide analysis above. We compare the evolution of modal intensities ${\left|A_{n}\right|}^{2}$ along the propagation distance z, obtained from FDTD simulations, with the results from the coupled‐mode theory. After propagating over a finite distance, the optical field initially confined in waveguide 1 couples into waveguide 2 and waveguide 3. The coupled field evolves into hybrid eigenmodes, as illustrated in Figure 2e. The optical field distribution at z = 2 cm corresponds to the position indicated by the yellow dashed line, whereas the field distribution at z = 19 cm corresponds to the position marked by the pink dashed line. Both the theoretical and simulated results are presented in Figure 2f.

As seen in the figure, the theoretical results align well with the coupled‐mode model, and the simulated coupling coefficients match the theoretical predictions. Under near‐degenerate conditions, waveguides 2 and 3 no longer exhibit identical behavior, which contrasts with the fully degenerate case. Notably, in nonequilateral triangle configurations, the coupling between waveguides on the longer side becomes negligible, which simplifies the overall coupling dynamics.

### 2.2 Corner‐State Oscillations and Total‐Angular‐Momentum Reversal

The time evolution of a particle or field in quantum systems is dictated by the time‐dependent Schrödinger equation $i\partial_{t}|\psi \left(t\right)\rangle =H\left(t\right)|\psi \left(t\right)\rangle$, where H(t) is the system Hamiltonian. For time‐independent systems, the eigenvalues and eigenstates of H determine the system's stationary behavior. However, in periodically driven systems where H(t+T)=H(t), the temporal modulation introduces qualitatively new features that cannot be captured by static Hamiltonians.

In such systems, the time evolution over one full period T is described by the unitary operator as follows:

(7) $$\begin{array}{c} U\left(T\right)=T\;\exp\left[-i\int_{0}^{T}H\left(t^{\prime }\right)dt^{\prime }\right], \end{array}$$

where T denotes time ordering. This operator defines the stroboscopic evolution of the system and allows one to construct an effective time‐independent Hamiltonian $H_{F}$ via $U\left(T\right)=\exp\left(-iH_{F}T\right)$. The eigenstates of $H_{F}$ govern the long‐time periodic dynamics of the system.

The waveguide lattice is composed of 16 unit cells in the transverse plane, with each unit cell containing two waveguides, as shown in Figure 1b. To implement the four‐step driving protocol using straight waveguides, metallic separators are selectively inserted between neighboring waveguides to suppress the undesired coupling channels, whereas the waveguide pair assigned to interact in each step is left unblocked. In this way, each site couples only to one of its four nearest neighbors during each driving step (Figure 3a,b). FDTD simulations further confirm that a metallic separator placed between two waveguides effectively suppresses their coupling, whereas the surrounding separators have a negligible influence on the intended unblocked coupling channel (Supporting Information S1: Figure S8). A global dimerization [32] is introduced by letting the coupling strength in one of the four steps differ from the other three: the coupling strength is $c_{1}$ for steps 1, 2, and 4, whereas for step 3 the coupling strength is denoted by $c_{2}$ as shown in Figure 3a. The coupling is characterized by the dimensionless integrated coupling angle θ=∫c(z)dz, which reduces to θ=cL for a constant coupling coefficient over a coupling length L. In the present four‐mode coupling configuration, owing to the simultaneous j‐preserving and j‐converting coupling channels, θ=π/2 produces complete conversion from j to −j in the initially excited waveguide. When illuminated by an optical vortex, a single waveguide can support two optical vortices with orders j and −j in a strongly confined system. For example, in step 1, the coupling constants are set as ${c_{1}}^{\prime }=\kappa_{13}=\kappa_{14}=\kappa_{23}=\kappa_{24}$. The actual coupling coefficient is a complex number, expressed as ${c_{1}}^{\prime }=c_{1}e^{i\alpha_{1}}$, where the phase angle $\alpha_{1}$ is determined by the j‐dependent coupling characteristic.

**FIGURE 3:**
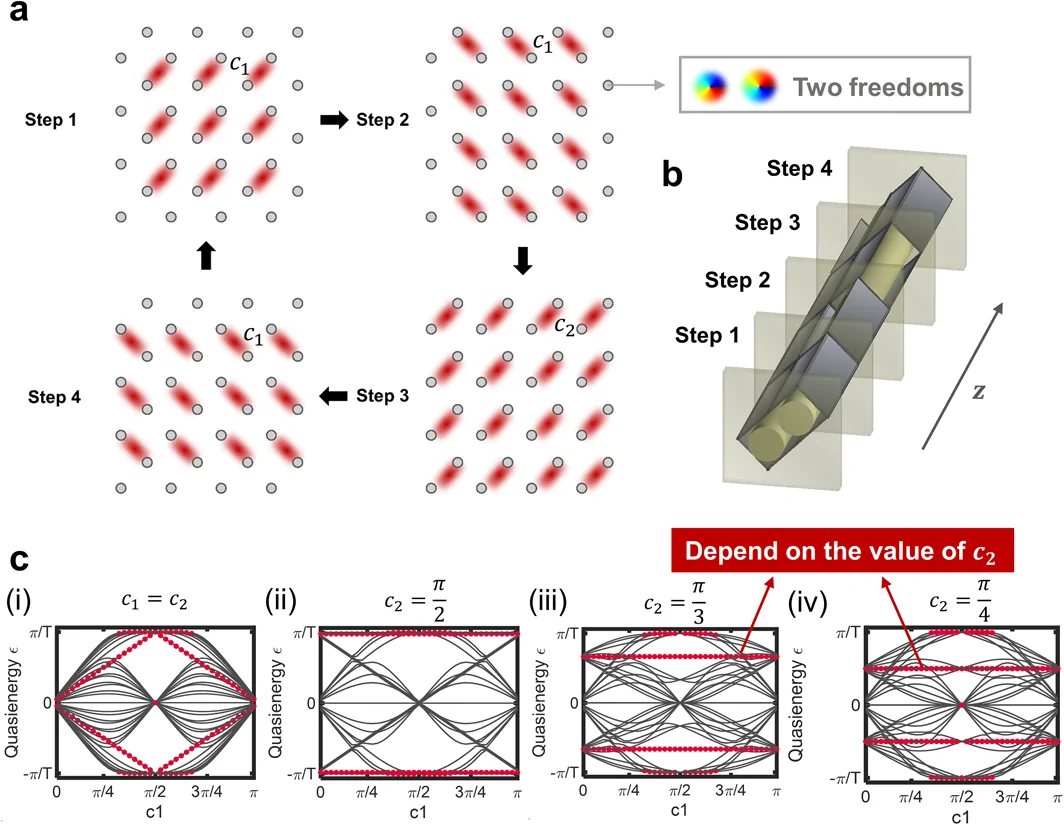
Coupling configuration at each step of the four‐step Floquet driving protocol and quasi‐energy spectrum. (a) The protocol consists of four distinct steps in which individual pairs of sites are allowed to interact. (b) Schematic of the unit cell of the waveguide array. (c) Numerically calculated quasi‐energy spectra of a finite structure of 32 waveguides under different coupling parameters.

The eigenspectra of finite structures with different coupling strengths $c_{1}$ and $c_{2}$ are presented in Figure 3c. For the case $c_{1}=c_{2}$, the quasi‐energy $\epsilon_{\text{corner}}$ of the corner states (marked by red circles) varies continuously with the coupling strength. In addition, corner states with a quasi‐energy of π are observed. Moreover, when $c_{2}=\frac{\pi }{2},\frac{\pi }{3},\frac{\pi }{4}$ and $c_{1}$ are fixed, the quasi‐energy of the corner state depends on the value of $c_{1}$. On this basis, the input state is given by the following equation:

(8) $$\begin{array}{c} |\psi \left(t\right)\rangle =e^{-i\epsilon_{\text{corner}}t}|\psi_{\text{corner}}\left(0\right)\rangle +e^{-i\epsilon_{\pi }t}|\psi_{\pi }\left(0\right)\rangle , \end{array}$$

As an example, we consider the case with $c_{1}=\frac{3\pi }{5}$ and $c_{2}=\frac{\pi }{4}$, where the corner‐state quasi‐energies are ±π and $\pm \frac{2\pi }{3}$. The corresponding quasi‐energy spectrum is shown in Figure 4b. When a corner state with order j is injected into the top‐left waveguide, the system exhibits a response with a period of $T_{\text{oscillation}}=4T$, returning to its initial state after four driving periods. Notably, after two periods, the waveguide at the corner undergoes an intrinsic conversion of the order from j to −j in Figure 4a, where the red bars represent the optical vortex with order j , and the blue bars represent the optical vortex with order −j. Similarly, for the case $c_{1}=\frac{3\pi }{5}$ and $c_{2}=\frac{\pi }{3}$, the corner‐state quasi‐energies are ±π and $\pm \frac{\pi }{2}$, as shown in Figure 4d. Under the same excitation condition, the system returns to its initial state after six driving periods, yielding $T_{\text{oscillation}}=6T$. A reversal of the order from j to −j occurs spontaneously at the corner waveguide after three periods (Figure 4c).

**FIGURE 4:**
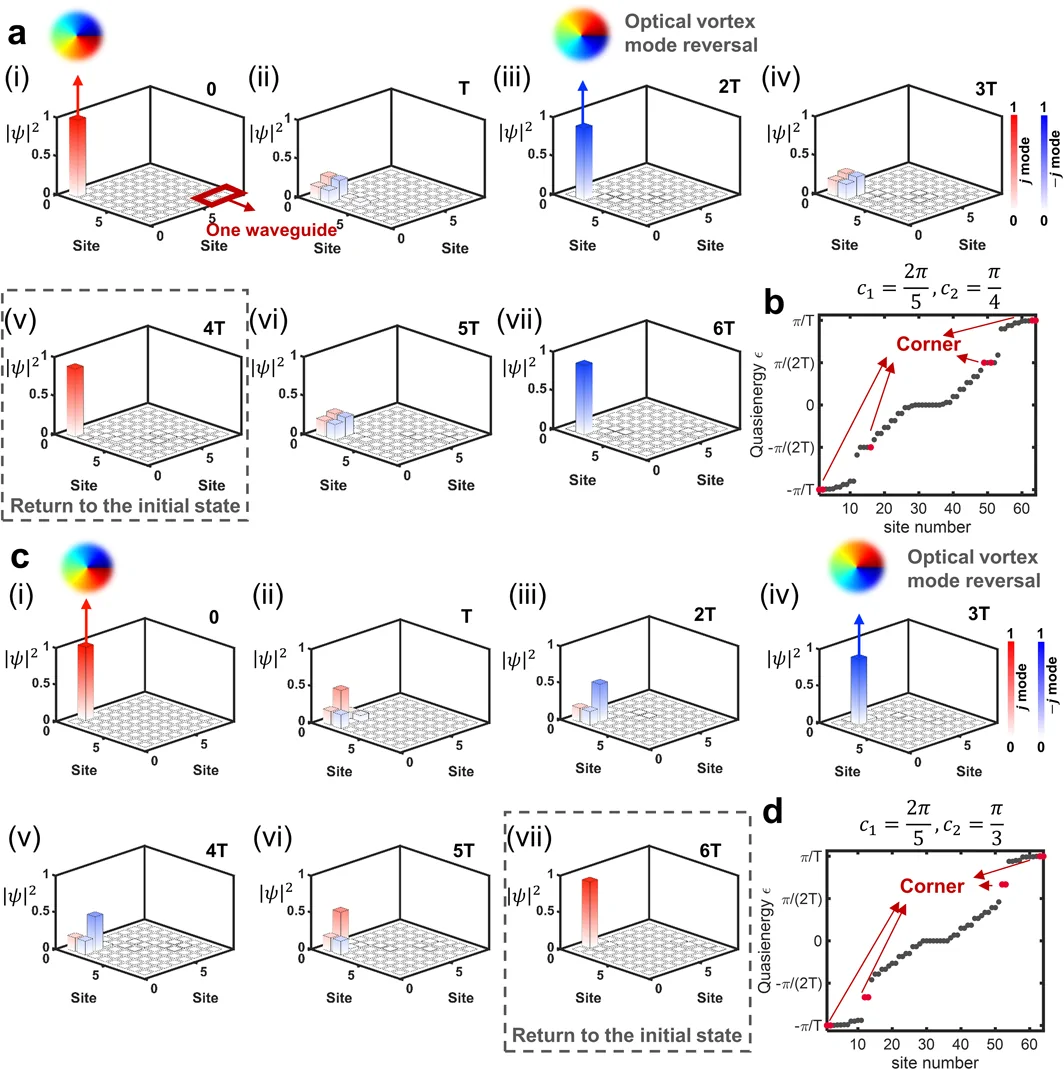
Reversal of the total angular momentum of optical‐vortex modes enabled by the oscillatory dynamics of the corner state. (a) Evolution of the optical vortex corner state for coupling parameters $c_{1}=\frac{3\pi }{5}$ and $c_{2}=\frac{\pi }{4}$. The state returns to its original field distribution after four driving periods, with a reversal of the total angular momentum occurring after two periods. (b) Quasi‐energy spectrum of the finite structure for coupling parameters $c_{1}=\frac{3\pi }{5}$ and $c_{2}=\frac{\pi }{4}$. (c) Evolution of the optical vortex corner state for coupling parameters $c_{1}=\frac{3\pi }{5}$ and $c_{2}=\frac{\pi }{3}$. The state returns to its original field distribution after six driving periods, with a reversal of the total angular momentum occurring after three periods. (d) Quasi‐energy spectrum of the finite structure for coupling parameters $c_{1}=\frac{3\pi }{5}$ and $c_{2}=\frac{\pi }{3}$.

### 2.3 Edge States With Coupled Optical Vortices of Different Orders

We consider two waveguides of different radii in air, with an azimuthal angle $\theta_{12}$ and a separation distance D as presented in Figure 5a. Suppose the incident light mode $E_{l,+1}$ enters waveguide 1, and the radius of waveguide 2 is adjusted such that mode $E_{m,+1}$ becomes phase‐matched with it, which means that $\beta_{l,+1}=\beta_{m,+1}$ (where m≠l). We define the Hilbert space as $\left\{E_{l,1},E_{-l,-1},E_{l,1},E_{-l,-1}\right\}\to \left\{E_{1},E_{2},E_{3},E_{4}\right\}$. The coupling matrix κ is given by the following equation:

(9) $$\begin{array}{c} \kappa =\left(\begin{array}{cccc} 0 & 0 & \kappa_{13}^{\left(0\right)}e^{-i\left(l-m\right)\theta_{12}} & \kappa_{14}^{\left(0\right)}e^{-i\left(l+m+2\right)\theta_{12}}\\ 0 & 0 & {\kappa_{14}^{\left(0\right)}}^{*}e^{i\left(l+m+2\right)\theta_{12}} & {\kappa_{13}^{\left(0\right)}}^{*}e^{i\left(l-m\right)\theta_{12}}\\ {\kappa_{13}^{\left(0\right)}}^{*}e^{i\left(l-m\right)\theta_{12}} & \kappa_{14}^{\left(0\right)}e^{-i\left(l+m+2\right)\theta_{12}} & 0 & 0\\ {\kappa_{14}^{\left(0\right)}}^{*}e^{i\left(l+m+2\right)\theta_{12}} & \kappa_{13}^{\left(0\right)}e^{-i\left(l-m\right)\theta_{12}} & 0 & 0 \end{array}\right). \end{array}$$

**FIGURE 5:**
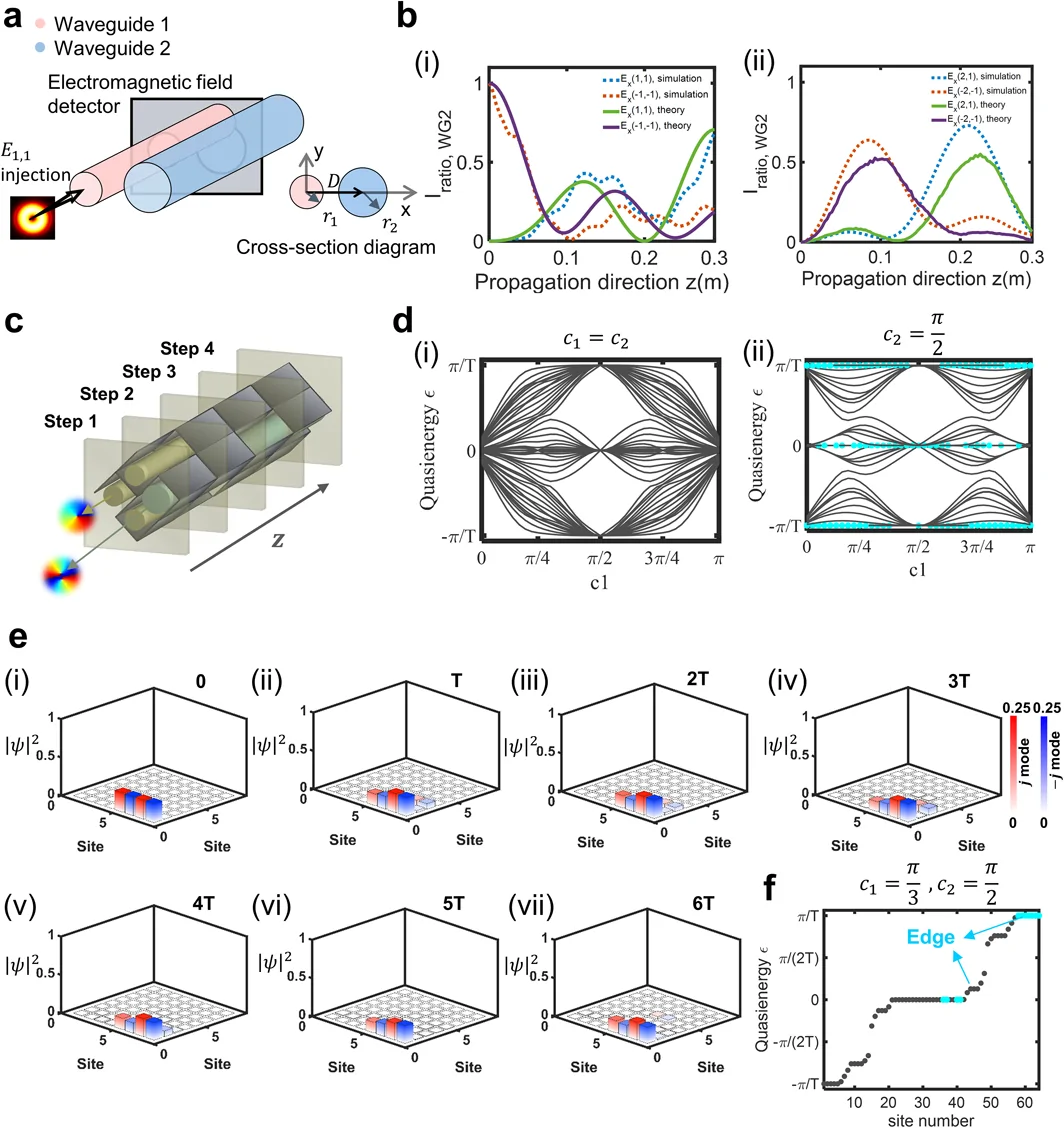
Two‐waveguide structure enabling coupling between different angular momentum characteristics and the quasi‐energy spectra. (a) Schematic of the two‐waveguide structure enabling coupling between distinct OAM and SAM orders. (b) Comparison of theoretical and simulated state evolution in waveguide 1 and waveguide 2. (c) Schematic of the unit cell of the waveguide array. (d) Numerically calculated quasi‐energy spectra of 32 waveguides under different coupling parameters. (e) Evolution of the optical‐vortex edge state for coupling parameters $c_{1}=\frac{\pi }{3}$ and $c_{2}=\frac{\pi }{2}$. After six driving periods, the state remains well localized at the system edge. (f) Quasi‐energy spectrum of the finite structure for coupling parameters $c_{1}=\frac{\pi }{3}$ and $c_{2}=\frac{\pi }{2}$.

The waveguide refractive index is $n_{1}=1.7$, the surrounding refractive index is $n_{0}=1.0$, and the wavelength of THz light in vacuum is λ=750μm. The radii of the two waveguides are $r_{1}=500\;\mu m$ and $r_{2}=690\;\mu m$, respectively, with a spacing of D=1300μm and an azimuthal angle θ=0. The input light is injected into waveguide 1 with mode $E_{-1,-1}$ corresponding to the Hilbert space basis $\left\{E_{1},E_{2},E_{3},E_{4}\right\}\to \left\{E_{1,1},E_{-1,-1},E_{1,1},E_{-1,-1}\right\}$. Because of the small waveguide size, near‐degenerate effects must be considered for low‐order modes (Supporting Information S1). We compare the evolution of ${\left|A_{n}\right|}^{2}$ with respect to z, as obtained from FDTD simulations, with the theoretical results, and the comparison results are shown in Figure 5b. The corresponding full width at half maximum is ${\Delta \lambda }_{\text{FWHM}}=130\;\mu m$ around the phase‐matching wavelength of 750μm, with the detailed analysis provided in Supporting Information S1: Figures S14 and S15.

We now consider a waveguide array with a cyclic driving protocol in which certain waveguides are replaced by ones with larger radii. These radii are chosen such that the effective mode index of the optical vortex with OAM order l= 2 in the large‐radius waveguides matches that of the optical vortex with OAM order l= 1 in the small‐radius waveguides, as illustrated in Figure 5c. The unit cell of the system now consists of four waveguides. The eigenenergy spectra of finite structures with varying coupling strengths $c_{1}$ and $c_{2}$ are shown in Figure 5d. When $c_{1}=c_{2}$, the quasi‐energy spectrum indicates the absence of corner and edge states. In contrast, when $c_{2}=\frac{\pi }{2}$ and $c_{1}$ is fixed, the system supports edge states with quasi‐energies at 0 and π. As an example, the case with $c_{1}=\frac{\pi }{3}$ and $c_{2}=\frac{\pi }{2}$, yields edge states with quasi‐energies at 0 and ±π (Figure 5d). When the light is injected along the edge of the waveguide lattice, it remains confined to the edge throughout propagation over several driving periods, as shown in Figure 5e.

## 3 Conclusion

We have demonstrated a photonic platform that supports complex coupling between optical vortices in strongly confined circular waveguide arrays. By constructing a Floquet driving protocol, where coupling configurations vary in each step, we realize corner and edge states, determined by the complex‐valued coupling coefficients. When only optical vortices with the same total angular momentum j are allowed to couple, we achieve corner states characterized by spontaneous flipping of the total‐angular‐momentum order j at half‐period intervals. When intermodal coupling between different j is introduced—facilitated by radius‐engineered phase matching between circular waveguides of different radii—we further demonstrate edge states. Our results show that the intrinsic phase structure of optical vortices, when integrated with dynamic modulation via a cyclic driving protocol, offers a robust route to explore self‐imaging dynamics with angular momentum encoding and periodic OAM inversion. The proposed terahertz structure is compatible with projection microstereolithography, whose micrometer‐scale fabrication resolution is well below the characteristic dimensions of the dielectric waveguides and metallic‐separator configuration considered here. For future experimental characterization, a vector network analyzer (VNA) combined with terahertz frequency multipliers could be used to perform frequency‐resolved measurements of the complex $S_{21}$ across the output facet. Its high dynamic range, extending to approximately −100 dB in the envisioned measurement configuration, may enable detection of the strongly attenuated output signal and reconstruction of the corresponding field distribution and OAM conversion. This work opens new possibilities for structured‐light control in both space and time, with potential applications in high‐capacity communications, quantum photonics, and reconfigurable on‐chip devices.

## Author Contributions

**Jinze Gao:** conceptualization, investigation, methodology, validation, visualization, software, data curation, formal analysis, writing – original draft. **Xin Xie:** writing – original draft, conceptualization, software, formal analysis. **Kun Liao:** writing – review and editing, resources, supervision, funding acquisition. **Xiaoyong Hu:** funding acquisition, writing – review and editing, supervision, resources, project administration.

## Funding

This work was supported by the National Key Research and Development Program of China under Grant No. 2024YFA1209204, the National Natural Science Foundation of China under Grant Nos. 12474322 and 12504428, the Quantum Science and Technology‐National Science and Technology Major Project under Grant No. 2021ZD0301500, Fundamental and Interdisciplinary Disciplines Breakthrough Plan of the Ministry of Education of China under Grant No. JYB2025XDXM106, and the Postdoctoral Fellowship Program and China Postdoctoral Science Foundation under Grant No. BX20250171.

## Supporting information


Supporting Information S1

## Data Availability

The data that support the findings of this study are available in the supplementary material of this article.
