# Supplementary material for: Conversion of Spin‐Orbit‐Coupled Optical Vortices via Complex Coupling
Source: Nanophotonics. 2026 Oct 23;15(20):e70337. doi: 10.1002/nap2.70337 (PMC13692709; doi:10.1002/nap2.70337)
Supplement: Supplementary file 1 [file NAP2-15-e70337-s001.pdf]

Supplementary Information for

Conversion of Spin-Orbit-coupled Optical Vortices via Complex Coupling

Jinze Gao[1], Xin Xie[1], Kun Liao[1*], Xiaoyong Hu[1,2,3,4*]

[1]State Key Laboratory for Mesoscopic Physics & Department of Physics, Collaborative Innovation Center of Quantum Matter & Frontiers Science Center for Nano-optoelectronics, Peking University, Beijing 100871, China

[2]Key Laboratory for Advanced Optoelectronic Integrated Chips of Jiangsu Province,

Peking University Yangtze Delta Institute of Optoelectronics, Nantong, Jiangsu 226010, China.

[3]Collaborative Innovation Center of Extreme Optics, Shanxi University, Taiyuan, Shanxi 030006, China

[4]Hefei National Laboratory, Hefei 230088,China

[*]E-mail: kunliao@pku.edu.cn; xiaoyonghu@pku.edu.cn

Table of Contents

Supplementary Notes:

Supplementary Note 1 | Analysis of the eigenmodes within the waveguide.

Supplementary Note 2 | Expressions for the electric and magnetic field components of the modes within the waveguide.

Supplementary Note 3 | Coupling coefficients between different modes in adjacent waveguides.

Supplementary Note 4 | Near-degenerate effects in triple-waveguide system and two waveguides of different radii.

Supplementary Note 5 | Comparison of coupling strength between identical and non-identical OAM mode orders.

Supplementary Note 6 | Verification of the topological nature of the corner state and edge state.

Supplementary Note 7 | Terahertz characterization of RG resin using terahertz time-domain spectroscopy (THz-TDS).

Supplementary Note 8 | Longitudinal scaling in the ideal Floquet model.

Supplementary Note 9 | $\boldsymbol{j} \rightarrow -\boldsymbol{j}$ conversion efficiencies at representative propagation distances.

Supplementary Note 10 | Preservation of the coupling mechanism with metallic separators.

Supplementary Note 11 | Bending-based implementation of the four-step Floquet protocol.

Supplementary Note 12 | Analysis of spin in strongly confined mode.

Supplementary Note 13 | Sample fabrication and practical implementation.

Supplementary Note 14 | Influence of material loss on the Floquet dynamics.

Supplementary Note 15 | Conversion efficiency between vortex modes of different orders.

Supplementary Figures:

Fig. S1 | Simulated electric field magnitude and phase distribution (x-polarized component)

Fig. S2 | The theoretical results for the magnitude and phase of the coupling coefficients.

Fig. S3 | Evolution of the bulk state under coupling parameters $\boldsymbol{c_1} = \frac{3\pi}{5}$ and $\boldsymbol{c_2} = \frac{\pi}{4}$.

Fig. S4 | Quasi-energy spectra under distinct coupling conditions.

Fig. S5 | Experimental setups of the THz-TDS characterization of RG resin films.

Fig. S6 | Terahertz characterization of RG resin films.

Fig. S7 | Structural implementation of the four-step Floquet lattice using dielectric waveguides and metallic coupling barriers.

Fig. S8 | Selective control of inter-waveguide coupling using metallic separators.

Fig. S9 | Quantitative evaluation of bending-induced loss and mode distortion.

Fig. S10 | Loss-induced Floquet quasi-energy linewidth and experimentally accessible propagation range.

Fig. S11 | Quasi-energy spectra for different relative coupling amplitudes between OAM modes.

Fig. S12 | Dependence of the propagation-constant mismatch on the FDE simulation resolution.

Fig. S13 | Influence of the propagation-constant mismatch on the coupling dynamics.

Fig. S14 | Waveguide dispersion and wavelength-dependent propagation-constant mismatch for the cross-OAM coupling scheme.

Fig. S15 | Influence of propagation-constant mismatch on the cross-OAM coupling dynamics.

Supplementary Table:

Table S1 | j → -j conversion efficiencies at representative propagation distances.

Supplementary Note 1 | Analysis of the eigenmodes within the waveguide.

By solving Maxwell's equations for an infinitely long circular waveguide, we obtain the electric field eigenmodes for a waveguide with core refractive index n_1, background refractive index n_0, and radius r.

$0 < r \leq a$:

$$E_n^x = 2A\beta \frac{a}{u}\left[\frac{(1-\xi)}{2} J_{n-1}\left(\frac{u}{a}r\right)\cos(n\theta - \theta + \psi + \beta z - \omega t) - \frac{(1+\xi)}{2} J_{n+1}\left(\frac{u}{a}r\right)\cos(n\theta + \theta + \psi + \beta z - \omega t)\right]$$

$$E_n^y = 2A\beta \frac{a}{u}\left[-\frac{(1-\xi)}{2} J_{n-1}\left(\frac{u}{a}r\right)\sin(n\theta - \theta + \psi + \beta z - \omega t) - \frac{(1+\xi)}{2} J_{n+1}\left(\frac{u}{a}r\right)\sin(n\theta + \theta + \psi + \beta z - \omega t)\right]$$

$$E_n^z = 2AJ_n\left(\frac{u}{a}r\right)\sin(n\theta + \psi + \beta z - \omega t) \tag{1}$$

$r > a$:

$$E_n^x = 2A\beta \frac{aJ_n(u)}{wK_n(w)}\left[\frac{(1-\xi)}{2} K_{n-1}\left(\frac{w}{a}r\right)\cos(n\theta - \theta + \psi + \beta z - \omega t) + \frac{(1+\xi)}{2} K_{n+1}\left(\frac{w}{a}r\right)\cos(n\theta + \theta + \psi + \beta z - \omega t)\right]$$

$$E_n^y = 2A\beta \frac{aJ_n(u)}{wK_n(w)}\left[-\frac{(1-\xi)}{2} K_{n-1}\left(\frac{w}{a}r\right)\sin(n\theta - \theta + \psi + \beta z - \omega t) + \frac{(1+\xi)}{2} K_{n+1}\left(\frac{w}{a}r\right)\sin(n\theta + \theta + \psi + \beta z - \omega t)\right]$$

$$E_n^z = -2A\frac{J_n(u)}{K_n(w)} K_n\left(\frac{w}{a}r\right)\sin(n\theta + \psi + \beta z - \omega t) \tag{2}$$

Here, E_n^x, E_n^y, and E_n^z represent the electric field components of the eigenmode E_n in the x, y and z directions, respectively. The coefficients $(1-\xi)/2$ and $(1+\xi)/2$ are used to separate the electric field into two parts:

$$E_n^x = \frac{(1-\xi)}{2} E_{n-1,1}^x + \frac{(1+\xi)}{2} E_{n+1,-1}^x \tag{3}$$

$$E_n^y = \frac{(1-\xi)}{2} E_{n-1,1}^y + \frac{(1+\xi)}{2} E_{n+1,-1}^y \tag{4}$$

$$E_n^z = \frac{(1-\xi)}{2} E_{n-1,1}^z + \frac{(1+\xi)}{2} E_{n+1,-1}^z \tag{5}$$

$$(E_n^z = E_{n-1,1}^z = E_{n+1,-1}^z)$$

Let the basis vectors of the Cartesian coordinate system be $\{e_x, e_y, e_z\}$. We define the electric field components as $E_{n-1,1} = E_{n-1}^x e_x + E_{n-1,1}^y e_y + E_{n-1,1}^z e_z$ and $E_{n+1,-1} = E_{n+1,-1}^x e_x + E_{n+1,-1}^y e_y + E_{n+1,-1}^z e_z$. Then, the total electric field E_n can be written as a linear combination of the two fields: $E_n = \frac{(1-\xi)}{2} E_{n-1,1} + \frac{(1+\xi)}{2} E_{n+1,-1}$. Although $E_{n-1,1}, E_{n+1,-1}$ are not eigenmodes of the circular waveguide, we can still analyze their circular-polarization components and azimuthal phase structures. Specifically, $E_{n-1,1}$ corresponds to the transverse circular-basis component with $\sigma = +1$, and $E_{n+1,-1}$ corresponds to the transverse circular-basis component with $\sigma = -1$. Here, $\sigma = \pm 1$ denotes the circular-spin basis index rather than the actual spin angular momentum of the full-vector mode. We identify $E_{n-1,1}$ as an OAM light with $j = n, l = n-1$, and $\sigma = +1$, $E_{n+1,-1}$ as an OAM light with $j = n, l = n+1$, and $\sigma = -1$. Thus, the full-vector waveguide eigenmode has a definite axial total-angular-momentum index $j = n$, formed by a superposition of left- and right-handed polarized components.

Using the same approach, the magnetic field can also be divided into two parts. Combined with the electric field, they form the corresponding full-vector electromagnetic circular-basis components characterized by $\sigma = \pm 1$.

By examining the phase distribution of E_x, we observe that when the OAM index l and the circular-spin basis index σ have the same sign, the field E_x closely resembles the phase distribution of right-handed (or left-handed) circularly polarized light. In this case, the parameter $\xi_1 \approx -1$ $(or\ \xi_1 \approx 1)$, and the total electric field approximately satisfies:

$$E_{j(1)} = E_{j-1,+1} + \left(\frac{1+\xi_1}{1-\xi_1}\right) E_{j-1,1} \left(or\ E_{j+1,-1} + \left(\frac{1-\xi_1}{1+\xi_1}\right) E_{j-1,1}\right)$$
$$\approx E_{j-1,1} \left(or\ E_{j+1,-1}\right) \tag{6}$$

When the OAM index l and the circular-spin basis index σ have opposite signs, the electric field component E_x appears as a mixture of the corresponding circularly polarized light and a weaker component of the opposite polarization. In this case, ξ_2 is no longer approximately ±1. However, the mode can still be considered mainly composed of the dominant circularly polarized component, while the other acts as a small perturbation.

$$E_{j(2)} = E_{j+1,-1} + \left(\frac{1-\xi_2}{1+\xi_2}\right) E_{j-1,1} \left(or\ E_{j-1,+1} + \left(\frac{1+\xi_2}{1-\xi_2}\right) E_{j+1,-1}\right) \tag{7}$$

Let the coefficient of the perturbation term be uniformly denoted as $E_{j(\gamma)}$. Then, the total field can be formally expressed as:

$$E_{j(\gamma)} = E_{j-s_\gamma,s_\gamma} + \xi_{j,s_\gamma} E_{j+s_\gamma,-s_\gamma}, \gamma = 1,2 \tag{8}$$

Supplementary Note 2 | Expressions for the electric and magnetic field components of the modes within the waveguide.

We consider a single circular dielectric waveguide embedded in free space. The refractive index of the waveguide medium is n_1, and the background medium has refractive index n_0. The waveguide has a radius a. The electromagnetic field distribution of the optical mode in cylindrical coordinates (r, θ, z) is given by the following components:

a. core region $(0 \leq r \leq a)$:

$$E^r = A\beta \frac{a}{u}\left[\frac{(1-\xi)}{2} J_{n-1}\left(\frac{u}{a}r\right) - \frac{(1+\xi)}{2} J_{n+1}\left(\frac{u}{a}r\right)\right] cos(n\theta + \psi)\, e^{i\beta z - i\omega t} + c.c. \tag{9}$$

$$E^\theta = -A\beta \frac{a}{u}\left[\frac{(1-\xi)}{2} J_{n-1}\left(\frac{u}{a}r\right) + \frac{(1+\xi)}{2} J_{n+1}\left(\frac{u}{a}r\right)\right] \sin(n\theta + \psi)\, e^{i\beta z - i\omega t} + c.c. \tag{10}$$

$$E^z = -iAJ_n\left(\frac{u}{a}r\right) \cos(n\theta + \psi)\, e^{i\beta z - i\omega t} + c.c. \tag{11}$$

$$H^r = A\omega\epsilon_0 n_1^2 \frac{a}{u}\left[\frac{(1-\xi_1)}{2} J_{n-1}\left(\frac{u}{a}r\right) + \frac{(1+\xi_1)}{2} J_{n+1}\left(\frac{u}{a}r\right)\right] \sin(n\theta + \psi)\, e^{i\beta z - i\omega t} + c.c. \tag{12}$$

$$H^\theta = A\omega\epsilon_0 n_1^2 \frac{a}{u}\left[\frac{(1-\xi_1)}{2} J_{n-1}\left(\frac{u}{a}r\right) - \frac{(1+\xi_1)}{2} J_{n+1}\left(\frac{u}{a}r\right)\right] \cos(n\theta + \psi)\, e^{i\beta z - i\omega t} + c.c. \tag{13}$$

$$H^z = iA\frac{\beta\xi}{\omega\mu_0}J_n\left(\frac{u}{a}r\right)\sin(n\theta+\psi)\,e^{i\beta z-i\omega t}+c.c. \tag{14}$$

b. cladding region($r \geq a$):

$$E^r = A\beta\frac{aJ_n(u)}{wK_n(w)}\left[\frac{(1-\xi)}{2}K_{n-1}\left(\frac{w}{a}r\right)+\frac{(1+\xi)}{2}K_{n+1}\left(\frac{w}{a}r\right)\right]\cos(n\theta+\psi)\,e^{i\beta z-i\omega t}+c.c. \tag{15}$$

$$E^\theta = -A\beta\frac{aJ_n(u)}{wK_n(w)}\left[\frac{(1-\xi)}{2}K_{n-1}\left(\frac{w}{a}r\right)-\frac{(1+\xi)}{2}K_{n+1}\left(\frac{w}{a}r\right)\right]\sin(n\theta+\psi)\,e^{i\beta z-i\omega t}+c.c. \tag{16}$$

$$E^z = -iA\frac{J_n(u)}{K_n(w)}K_n\left(\frac{u}{a}r\right)\cos(n\theta+\psi)\,e^{i\beta z-i\omega t}+c.c. \tag{17}$$

$$H^r = A\omega\epsilon_0 n_0^2\frac{aJ_n(u)}{wK_n(w)}\left[\frac{(1-\xi_0)}{2}K_{n-1}\left(\frac{w}{a}r\right)-\frac{(1+\xi_0)}{2}K_{n+1}\left(\frac{u}{a}r\right)\right]\sin(n\theta+\psi)\,e^{i\beta z-i\omega t}+c.c. \tag{18}$$

$$H^\theta = A\omega\epsilon_0 n_0^2\frac{aJ_n(u)}{wK_n(w)}\left[\frac{(1-\xi_0)}{2}K_{n-1}\left(\frac{w}{a}r\right)+\frac{(1+\xi_0)}{2}K_{n+1}\left(\frac{w}{a}r\right)\right]\cos(n\theta+\psi)\,e^{i\beta z-i\omega t}+c.c. \tag{19}$$

$$H^z = iA\frac{\beta\xi}{\omega\mu_0}\frac{J_n(u)}{K_n(w)}K_n\left(\frac{w}{a}r\right)\sin(n\theta+\psi)\,e^{i\beta z-i\omega t}+c.c. \tag{20}$$

Where β is the effective longitudinal wave vector, $\xi = n(u^{-2}+w^{-2})/\left[\frac{J_n'(u)}{uJ_n(u)}+\frac{K_n'(w)}{wK_n(w)}\right]$, $\xi_1 = \frac{\beta^2}{k_0^2n_1^2}\xi$, $\xi_0 = \frac{\beta^2}{k_0^2n_0^2}\xi$, $A = 1/\int_{-\infty}^{\infty}\int_{-\infty}^{\infty} e_z\cdot(\tilde{E}^*\times\tilde{H}+\tilde{E}\times\tilde{H}^*)dxdy$ is normalization constant based on energy flow.

The normalized eigenmode is denoted $\{E_n^{tot}(r,\theta,z;t,n,\psi), H_n^{tot}(r,\theta,z;t,n,\psi)\}$. The circularly polarized OAM mode can be expressed as:

$$E_n = E_n^{tot}(r,\theta,z;t,n,\psi)+E_n^{tot}\left(r,\theta,z;t-\frac{\pi}{2\omega},n,\psi-\frac{\pi}{2}\right) \tag{21}$$

Transforming to Cartesian components E^x, E^y, E^z, the field expressions inside and outside the waveguide become:

$0 < r \leq a$:

$$E_n^x = 2A\beta\frac{a}{u}\left[\frac{(1-\xi)}{2}J_{n-1}\left(\frac{u}{a}r\right)\cos(n\theta-\theta+\psi+\beta z-\omega t)\right.$$

$$\left.-\frac{(1+\xi)}{2}J_{n+1}\left(\frac{u}{a}r\right)\cos(n\theta+\theta+\psi+\beta z-\omega t)\right]$$

$$E_n^y = 2A\beta\frac{a}{u}\left[-\frac{(1-\xi)}{2}J_{n-1}\left(\frac{u}{a}r\right)\sin(n\theta-\theta+\psi+\beta z-\omega t)\right.$$

$$\left.-\frac{(1+\xi)}{2}J_{n+1}\left(\frac{u}{a}r\right)\sin(n\theta+\theta+\psi+\beta z-\omega t)\right]$$

$$E_n^z = 2AJ_n\left(\frac{u}{a}r\right)\sin(n\theta+\psi+\beta z-\omega t) \tag{22}$$

$r > a$:

$$E_n^x = 2A\beta\frac{aJ_n(u)}{wK_n(w)}\left[\frac{(1-\xi)}{2}K_{n-1}\left(\frac{u}{a}r\right)\cos(n\theta-\theta+\psi+\beta z-\omega t)\right.$$

$$\left.+\frac{(1+\xi)}{2}K_{n+1}\left(\frac{u}{a}r\right)\cos(n\theta+\theta+\psi+\beta z-\omega t)\right]$$

$$E_n^y = 2A\beta\frac{aJ_n(u)}{wK_n(w)}\left[-\frac{(1-\xi)}{2}K_{n-1}\left(\frac{u}{a}r\right)\sin(n\theta-\theta+\psi+\beta z-\omega t)\right.$$

$$\left.+\frac{(1+\xi)}{2}K_{n+1}\left(\frac{u}{a}r\right)\sin(n\theta+\theta+\psi+\beta z-\omega t)\right]$$

$$E_n^z = -2A\frac{J_n(u)}{K_n(w)}K_n\left(\frac{u}{a}r\right)\sin(n\theta+\psi+\beta z-\omega t) \tag{23}$$

Supplementary Note 3 | Coupling coefficients between different modes in adjacent waveguides.

We analyze the propagation of light in a system of multiple optical waveguides using classical coupled mode theory. Let the waveguide index be labeled by a ($a = 1,2,\ldots,M$). The normalized eigenmode of the $n-th$ mode in the $a-th$ waveguide is written as:

$$E_{an}(x,y,z,t) = E_{an}(x,y)e^{i\beta_{an}z-i\omega t} + c.c. = \tilde{E}_{an}(x,y,z,t) + c.c. \tag{24}$$

$$H_{an}(x,y,z,t) = H_{an}(x,y)e^{i\beta_{an}z-i\omega t} + c.c. = \tilde{H}_{an}(x,y,z,t) + c.c. \tag{25}$$

These modes satisfy Maxwell's equations in the medium with refractive index profile$N_a(x,y)$, which represents the $a-th$ waveguide alone:

$$\nabla\times\tilde{E}_{an} = i\omega\mu_0\tilde{H}_{an} \tag{26}$$

$$\nabla\times\tilde{H}_{an} = -i\omega\epsilon_0 N_a^2(x,y)\tilde{E}_{an} \tag{27}$$

Now consider the total electric and magnetic fields in the multi-waveguide system as a superposition of all individual modes, weighted by their slowly varying amplitudes

$$E(x,y,z,t) = \sum_{a,n} C_{an}(z)E_{an}(x,y)e^{i\beta_{an}z-i\omega t} + c.c. = \tilde{E}(x,y,z,t) + c.c. \tag{28}$$

$$H(x,y,z,t) = \sum_{a,n} C_{an}(z)H_{an}(x,y)e^{i\beta_{an}z-i\omega t} + c.c. = \tilde{H}(x,y,z,t) + c.c. \tag{29}$$

The total fields obey Maxwell's equations in the overall refractive index distribution $N(x,y)$ of the full waveguide system:

$$\nabla \times \tilde{E} = i\omega\mu_0\tilde{H} \tag{30}$$

$$\nabla \times \tilde{H} = -i\omega\epsilon_0 N^2(x,y)\tilde{E} \tag{31}$$

Then

$$\sum_{a,n} \left(e_z \times \tilde{E}_{an}\right)\frac{dC_{an}}{dz} = 0 \tag{32}$$

$$\sum_{a,n} \left[\left(e_z \times \tilde{H}_{an}\right)\frac{dC_{an}}{dz} + i\omega\epsilon_0(N^2 - N_a^2)\tilde{E}_{an}C_{an}\right] = 0 \tag{33}$$

$$\kappa_{an,bm}(z) = \frac{-\omega\epsilon_0 \int_{-\infty}^{\infty}\int_{-\infty}^{\infty}(N^2 - N_b^2)\tilde{E}_{an}^* \cdot \tilde{E}_{bm}dxdy}{\int_{-\infty}^{\infty}\int_{-\infty}^{\infty} e_z \cdot \left(\tilde{E}_{an}^* \times \tilde{H}_{an} + \tilde{E}_{an} \times \tilde{H}_{an}^*\right)dxdy} \tag{34}$$

$$c_{an,bm}(z) = \frac{\int_{-\infty}^{\infty}\int_{-\infty}^{\infty} e_z \cdot \left(\tilde{E}_{an}^* \times \tilde{H}_{bm} + \tilde{E}_{an} \times \tilde{H}_{bm}^*\right)dxdy}{\int_{-\infty}^{\infty}\int_{-\infty}^{\infty} e_z \cdot \left(\tilde{E}_{an}^* \times \tilde{H}_{an} + \tilde{E}_{an} \times \tilde{H}_{an}^*\right)dxdy} \tag{35}$$

The formula is simplified to the following form:

$$\sum_{b,m} ic_{an,bm}(z)\frac{dC_{bm}}{dz} + \kappa_{an,bm}(z)C_{bm} = 0 \tag{36}$$

For light propagating in a waveguide, the light intensity inside the waveguide is much greater than the light intensity outside the waveguide. It can be approximated that $c_{an,bm}(z) = \delta_{an,bm}$, so the final coupling mode equation is

$$i\frac{dC_{an}}{dz} = \sum_{b,m} \kappa_{an,bm}(z)C_{bm} \tag{37}$$

The expression for the coupling coefficient $\kappa_{an,bm}$ is given by:

$$\kappa_{an,bm} = \frac{-\omega\epsilon_0 \int_{-\infty}^{\infty}\int_{-\infty}^{\infty}(N^2 - N_b^2)E_{an}^*(x,y)\cdot E_{bm}(x,y)dxdy}{\int_{-\infty}^{\infty}\int_{-\infty}^{\infty} e_z \cdot [E_{an}^*(x,y)\times H_{an}(x,y) + E_{an}(x,y)\times H_{an}^*(x,y)]\, dxdy} \quad (38)$$

Due to energy flux normalization, for each normalized mode, the denominator, $S = \int_{-\infty}^{\infty}\int_{-\infty}^{\infty} e_z \cdot [E_{an}^*(x,y)\times H_{an}(x,y) + E_{an}(x,y)\times H_{an}^*(x,y)]\, dxdy$ is a constant. For the numerator, involving the term $\int_{-\infty}^{\infty}\int_{-\infty}^{\infty}(N^2(x,y) - N_b^2(x,y))E_{an}^*(x,y)\cdot E_{bm}(x,y)dxdy$, in a multi-waveguide system, due to the spatial properties of the refractive index distribution, if $a \neq b$, it simplifies to

$$\int_{-\infty}^{\infty}\int_{-\infty}^{\infty}(N^2(x,y) - N_b^2(x,y))E_{an}^*(x,y)\cdot E_{bm}(x,y)dxdy$$

$$= \sum_{d\neq b}\iint_{S_d}(n_1^2 - n_0^2)E_{an}^*(x,y)\cdot E_{bm}(x,y)dxdy$$

$$= \iint_{S_a}(n_1^2 - n_0^2)E_{an}^*(x,y)\cdot E_{bm}(x,y)dxdy \quad (39)$$

where S_a is the cross-sectional region of waveguide a in the xy-plane. Therefore, the coupling only depends on the field overlap within waveguide a, and the expression becomes:

$$\kappa_{an,bm} = \frac{-\omega\epsilon_0}{S}\iint_{S_a}(n_1^2 - n_0^2)E_{an}^*(x,y)\cdot E_{bm}(x,y)dxdy \quad (40)$$

This expression does not include the effect of waveguides other than a and b, implying that the coupling coefficient in a multi-waveguide system is the same as that in a dual-waveguide system. Moreover, the relation $\kappa_{an,bm} = \kappa_{bm,an}^*$ holds.

If $a = b$ $(a \neq d)$, the term $\kappa_{an,bm} \ll \kappa_{an,dm}$, and can be approximated as zero. This means that the coupling coefficient between two modes of the same waveguide is assumed to be zero in a multi-waveguide system.

Supplementary Note 4 | Near-degenerate effects in triple-waveguide system and two waveguides of different radii.

Triple-waveguide system

If the modes are near-degenerate, the longitudinal wave vectors become $\beta, \beta + d\beta$. Let $A_{1,3,5} = C_{1,3,5}, A_{2,4,6} = C_{2,4,6} e^{i(d\beta)z}$. Then the coupled mode equation becomes $i\frac{dA_n}{dz} = \sum_m \kappa_{n,m}(d\beta) A_m$. The corrected coupling matrix under near-degenerate conditions is:

$$\kappa(d\beta) = \kappa - O^{-1} \begin{pmatrix} 0 & 0 & 0 & 0 & 0 & 0 \\ 0 & d\beta & 0 & 0 & 0 & 0 \\ 0 & 0 & 0 & 0 & 0 & 0 \\ 0 & 0 & 0 & d\beta & 0 & 0 \\ 0 & 0 & 0 & 0 & 0 & 0 \\ 0 & 0 & 0 & 0 & 0 & d\beta \end{pmatrix} O \tag{41}$$

where the transformation matrix O is:

$$O = \begin{pmatrix} 1 & 1 & 0 & 0 & 0 & 0 \\ i & -i & 0 & 0 & 0 & 0 \\ 0 & 0 & 1 & 1 & 0 & 0 \\ 0 & 0 & i & -i & 0 & 0 \\ 0 & 0 & 0 & 0 & 1 & 1 \\ 0 & 0 & 0 & 0 & i & -i \end{pmatrix} \tag{42}$$

The coupled equations can be solved analytically:

$$A_n(z) = \sum_s A_{s,n} e^{-i\kappa_s z} \tag{43}$$

where $\{\kappa_s, A_{s,an}\}$ are the eigenvalues and eigenvectors of $\kappa(d\beta)$.

Two waveguides of different radii

In the near-degenerate case, the same correction method described previously can be applied. The corrected coupling matrix is:

$$\kappa(d\beta_1, d\beta_2) = \kappa - O^{-1} \begin{pmatrix} 0 & 0 & 0 & 0 \\ 0 & d\beta_1 & 0 & 0 \\ 0 & 0 & d\beta_2 & 0 \\ 0 & 0 & 0 & d\beta_2 \end{pmatrix} O \tag{44}$$

Wavelength-dependent phase matching and operational bandwidth

For coupling between vortex modes of different orders supported by waveguides of different radii, the phase-matching condition is wavelength dependent because the two modes exhibit different waveguide dispersions. We define the wavelength-dependent propagation-constant mismatch as $\Delta\beta = \beta_1 - \beta_2$, where β_1 and β_2 are the propagation constants of the two modes participating in the cross-angular-

momentum coupling. At the designed operating wavelength, the two modes satisfy approximately $\Delta\beta = 0$, whereas wavelength detuning produces a finite phase mismatch, as shown in Supplementary Fig. S14.

The finite mismatch is incorporated into the coupled-mode Hamiltonian through the corresponding diagonal propagation terms. We then calculate the modal evolution while systematically varying $\Delta\beta$. Because the two vortex-mode components in waveguide 2 reach their conversion maxima at different propagation distances, the maximum conversion efficiency of the selected target mode is used to quantify the coupling resonance. The half-maximum condition is defined as $\frac{A_4^2(\Delta\beta)}{A_4^2(0)} = 0.5$, where $A_4^2(0)$ is the maximum conversion efficiency under ideal phase matching. Using the wavelength-dependent dispersion obtained from the FDE eigenmode calculations, the corresponding half-width in propagation-constant mismatch can be converted into a wavelength detuning. Assuming an approximately symmetric response around the phase-matching wavelength, the resulting full width at half maximum of the cross-angular-momentum coupling resonance is approximately 130 um, corresponding to a frequency bandwidth of approximately 70 GHz. Supplementary Fig. S15 compares the propagation dynamics under ideal phase matching and at the half-maximum mismatch and shows that wavelength detuning modifies both the attainable conversion efficiency and the propagation distance at which the conversion maximum occurs.

Supplementary Note 5 | Comparison of coupling strength between identical and non-identical OAM mode orders.

$$\kappa_{an,bm} = \frac{-\omega\epsilon_0 \int_{-\infty}^{\infty}\int_{-\infty}^{\infty}(N^2 - N_b^2)E_{an}^*(x,y)\cdot E_{bm}(x,y)dxdy}{\int_{-\infty}^{\infty}\int_{-\infty}^{\infty} e_z\cdot[E_{an}^*(x,y)\times H_{an}(x,y) + E_{an}(x,y)\times H_{an}^*(x,y)]\,dxdy} \quad (45)$$

For the coupling configuration discussed previously, the absolute value of the ratio $\left|\frac{\kappa_{13}}{\kappa_{14}}\right|$ increases with the inter-waveguide separation d. Since the initial magnitude of κ_{13} is smaller than that of κ_{14}, there exists a specific distance at which the two coupling strengths become equal in magnitude.

Supplementary Note 6 | Verification of the topological nature of the corner state and edge state.

To verify the topological nature of the corner state under the cyclic driving protocol, random perturbations are introduced into the system. For example, under the parameters $c_1 = \frac{3\pi}{5}$ and $c_2 = \frac{\pi}{3}$, 63 independent perturbations are applied to the inter-waveguide couplings. Based on the perturbed Hamiltonian, the quasi-energy spectrum is recalculated, revealing that the corner state at quasi-energy $\frac{2\pi}{3}$ remains robust, thereby confirming its topological protection (Fig S4).

In contrast, when similar perturbations are applied under identical or different parameter regimes, we observe that the corner state at zero quasi-energy, as well as certain edge states, may vanish. This suggests that such states are not topologically protected, but may arise from structural asymmetries or accidental degeneracies between decoupled subspaces, leading to the formation of bound states in the continuum (BICs).

Supplementary Note 7 | Terahertz characterization of RG resin using terahertz time-domain spectroscopy (THz-TDS).

The terahertz optical properties of the RG resin were characterized using a THz-TDS system. As schematically illustrated in Fig. S5, a femtosecond laser is divided into pump and probe paths for THz generation and coherent detection. The generated THz radiation is transmitted through the RG resin film, and the transmitted time-domain electric field was recorded and compared with the corresponding reference signal. Fourier transformation of the time-domain waveforms provided the frequency-dependent amplitude and phase, from which the complex refractive index and absorption coefficient of the resin are extracted.

As shown in Fig. S6, the real part of the refractive index gradually decreases with increasing frequency, whereas the imaginary part and absorption coefficient increase over the measured THz range. The comparison with several other commercial resins further shows that RG exhibits the lowest absorption among the investigated materials

over most of the measured frequency range. These experimentally characterized optical constants are used to evaluate the influence of material absorption on the proposed THz waveguide and Floquet dynamics.

Supplementary Note 8 | Longitudinal scaling in the ideal Floquet model.

In the ideal symmetric model, the coupling amplitudes of the relevant j-preserving and j-converting channels are both taken to be the same real value c; the complex phase factor is retained separately in the coupling matrix. We define the integrated coupling angle as

$$\theta(L) = \int_0^L c(z)\,dz. \tag{46}$$

For a coupling section in which c is approximately constant, $\theta = cL$. Thus the theoretical coupling parameter is an integrated coupling angle rather than a criterion based on complete power transfer between the two physical waveguides.

For an input state $|A,+j\rangle$, unitary evolution of the ideal four-mode coupling block gives the modal amplitudes

$$\begin{gathered} a_{A,+j} = cos2\theta, a_{A,-j} = \Delta * \ sin2\theta, \\ a_{B,+j} = -isin\theta cos\theta, a_{B,-j} = i\Delta * sin\theta cos\theta. \end{gathered} \tag{47}$$

Taking the modulus squared of each amplitude gives the corresponding modal intensities:

$$\begin{gathered} P_{A,+j} = cos4\theta \\ P_{A,-j} = sin4\theta \\ P_{B,+j} = P_{B,-j} = sin2\theta\ cos2\theta. \end{gathered} \tag{48}$$

The four modal intensities satisfy $P_{A,+j} + P_{A,-j} + P_{B,+j} + P_{B,-j} = 1$. At $\theta = \pi/2$, these expressions reduce to $P_{A,-j} = 1$, while $P_{A,+j} = P_{B,+j} = P_{B,-j} = 0$. Therefore, in the ideal four-mode system, the condition $\theta = \pi/2$ corresponds to complete $+j \rightarrow -j$ conversion in the initially excited waveguide, rather than complete transfer from waveguide A to waveguide B. The physical $\pi/2$ coupling length is consequently defined by

$$cL_{\pi/2} = \pi/2, L_{\pi/2} = \pi/(2c). \tag{49}$$

The physical length of each Floquet step is then obtained by matching the integrated coupling angle used in the theoretical model.

Supplementary Note 9 | j → -j conversion efficiencies at representative propagation distances.

To quantitatively characterize the conversion between the j and $-j$ vortex modes, we evaluate both the local and global conversion efficiencies during the Floquet evolution. The local conversion efficiency at the initially excited site is defined as

$$\eta_{local} = \frac{P_{-j,site}}{P_{j,site} + P_{-j,site}}, \tag{50}$$

where $P_{j,site}$ and $P_{-j,site}$ denote the powers carried by the j and $-j$ modes, respectively, at the initially excited site.

The global conversion efficiency is defined as

$$\eta_{global} = \frac{P_{-j,all}}{P_{total}}, \tag{51}$$

where $P_{-j,all}$ is the total power carried by the $-j$ modes throughout the lattice. As summarized in Supplementary Table S1, the local conversion efficiencies remain between 99.214% and 99.962%, while the corresponding global conversion efficiencies range from 91.826% to 98.093% for the two representative coupling conditions. The conversion efficiencies do not reach exactly 100% because the initially excited single-site state is not an exact Floquet corner eigenstate. Although the dominant part of the input state projects onto the corner-state eigenmodes responsible for the $j \to -j$ conversion, a small residual component projects onto the bulk eigenstates. This bulk-state contribution undergoes different Floquet evolution and therefore prevents perfectly complete $j \to -j$ conversion, particularly when the conversion efficiency is evaluated globally over the entire lattice. Nevertheless, these results quantitatively demonstrate highly efficient conversion between the j and $-j$ vortex modes in the proposed Floquet lattice. These results show that the $j \to -j$ conversion at the initially

excited site is nearly complete, while high conversion efficiency is also maintained over the entire lattice.

Supplementary Note 10 | Preservation of the coupling mechanism with metallic separators.

For a pair of neighboring waveguides A and B, the coupling coefficient can be described within coupled-mode theory as

$$\kappa_{an,bm} = \frac{-\omega\epsilon_0}{S}\iint_{S_a}(n_1^2 - n_0^2)E_{an}^*(x,y)\cdot E_{bm}(x,y)dxdy\,, \tag{52}$$

as same as equation (40), where E_{an} and E_{bm} are the isolated-waveguide modal fields, S_a denotes the cross-sectional region of waveguide A. Thus, the A-B coupling is primarily governed by the evanescent-field overlap in their mutual coupling region.

In the metallic-separator implementation, no metallic separator is placed between the waveguide pair intended to remain coupled. The waveguide geometry, center-to-center separation, and dominant evanescent-overlap region between A and B therefore remain unchanged. Separators are instead introduced along the surrounding undesired coupling channels. Their influence on the modal fields in the A-B coupling region can be treated as a weak perturbation,

$$E_{an}^{metal} = E_{an} + \delta E_{an}, E_{bm}^{metal} = E_{bm} + \delta E_{bm} \tag{53}$$

which gives, to first order,

$$\kappa_{an,\,bm}^{metal} = \kappa_{an,bm} + \delta\kappa_{an,bm}, |\delta\kappa_{an,bm}| \ll |\kappa_{an,bm}| \tag{54}$$

Consequently, the intended unblocked coupling channel satisfies

$$\kappa_{an,\,bm}^{metal} \simeq \kappa_{an,bm} \tag{55}$$

By contrast, when a metallic separator is placed directly between an undesired waveguide pair, the metallic boundary strongly suppresses the tangential electric field and hence the evanescent-field overlap across that bond, reducing the corresponding coupling coefficient toward zero. In the present design, each metallic separator has a cross-sectional width of 50 μm and a transverse length of 1450 μm. The metallic

separators therefore selectively suppress the undesired coupling channels while approximately preserving the coupling mechanism and coupling strength of the intended unblocked waveguide pair.

Supplementary Note 11 | Bending-based implementation of the four-step Floquet protocol.

As an alternative implementation of the four-step Floquet driving protocol, the step-dependent coupling configuration can also be realized by smoothly bending the waveguides, following the general strategy used in waveguide-based Floquet lattices. In each driving step, the waveguide pairs assigned to interact are smoothly brought into closer proximity, thereby enhancing their coupling, while the other waveguide pairs remain sufficiently separated to suppress undesired coupling channels. Repeating the four coupling configurations along the propagation direction realizes the same four-step Floquet sequence considered in the ideal model.

To avoid abrupt changes in the propagation direction, the lateral displacement of each bending section is described by a smooth cosine trajectory,

$$x(z) = \frac{\Delta x}{2}\left[1 - cos\left(\frac{\pi z}{L_{bend}}\right)\right], \tag{56}$$

where Δx is the lateral displacement and L_{bend} is the longitudinal length of the bending section. The corresponding local bending radius is determined by the curvature of the trajectory

$$R(z) = \frac{\left[1 + \left(\frac{dx}{dz}\right)^2\right]^{\frac{3}{2}}}{\left|\frac{d^2x}{dz^2}\right|}, \tag{57}$$

with the minimum bending radius given by $R_{min} = \frac{2L_{bend}^2}{\pi^2 \Delta x}$. The detailed geometric parameters required for the four-step protocol, together with the FDTD evaluation of the corresponding bending loss, OAM-mode power preservation, and OAM fidelity, are provided in Supplementary Fig. S9.

Supplementary Note 12 | Analysis of spin in strongly confined mode.

To quantify the deviation from the scalar-SAM approximation, we explicitly evaluate the electromagnetic spin density from the complete electric and magnetic fields,

$$S(r) = \frac{1}{4\omega}\mathrm{Im}\left[\varepsilon(r)\overline{E} \times E + \mu(r)\overline{H} \times H\right]. \quad (58)$$

The corresponding energy-normalized mean spin per photon is

$$s_{\mathrm{avg}} = \frac{\omega \int S(r)\, dA}{\int W(r)\, dA}, \quad (59)$$

and its axial component is

$$s_{z,avg} = \frac{\omega \int S_z(r)\, dA}{\int W(r)\, dA}, \quad (60)$$

where $W(r)$ is the time-averaged electromagnetic energy density. The full-field calculation confirms that the local spin density contains nonzero transverse components and that the axial mean spin of the strongly confined modes is not exactly ±1. The two partner modes considered in this work have opposite spin-density textures and equal-magnitude, opposite energy-normalized axial spin, with $|s_{z,avg}| < \hbar$ owing to the strong confinement. Specifically, $s_{z,avg}(E_{1,1}) = 0.927\hbar, s_{z,avg}(E_{-1,-1}) = -0.927\hbar, s_{z,avg}(E_{2,1}) = 0.879\hbar$, and $s_{z,avg}(E_{-2,-1}) = -0.879\hbar$.

The deviation of the mean modal spin from the paraxial value does not alter the phase relation of the inter-waveguide complex coupling. The coupling coefficient is evaluated from the overlap of the complete vector electromagnetic modes and therefore does not require a spatially uniform scalar-SAM approximation. For two full-vector modes characterized by axial rotational eigenvalues j and j', rotation of the coupling geometry by an azimuthal angle θ transforms the coupling coefficient according to $\kappa_{jj'}(\theta) = e^{i(j-j')\theta}\kappa_{jj'}(0)$, up to the phase convention adopted for the modal basis. This relation follows from the rotational covariance of the complete vector eigenmodes. The longitudinal field components obey the same total rotational transformation as the transverse components and therefore modify the magnitude of the modal overlap but

do not invalidate its angular phase dependence. Consequently, the complex-coupling phase used in the Floquet model remains governed by the rotational indices of the full-vector modes even though the local spin density is spatially inhomogeneous and the cross-section-averaged spin is not restricted to ±1.

Supplementary Note 13 | Sample fabrication and practical implementation.

The proposed terahertz waveguide samples can be fabricated using projection micro stereolithography (PμSL), a DMD-assisted UV photopolymerization technique with a nominal spatial resolution of approximately 2 μm, which is much smaller than the characteristic waveguide dimensions of hundreds of micrometers. The cured resin provides sufficient mechanical rigidity to maintain the three-dimensional waveguide geometry. For the four-step coupling protocol, the dielectric waveguides remain straight, while metallic separators are introduced between selected neighboring waveguides to suppress the inactive coupling channels. The polymer waveguide structure can therefore be fabricated by PμSL and subsequently integrated with the metallic shielding structures, providing a practical route for implementing the proposed platform.

Supplementary Note 14 | Influence of material loss on the Floquet dynamics.

To evaluate the influence of material absorption on the Floquet dynamics, we incorporate the experimentally characterized propagation loss into the tight-binding model through a non-Hermitian propagation term. In the experimentally relevant isotropic-loss limit, the Hamiltonian during each driving step can be written as

$$H_{NH} = H - i\gamma I, \tag{61}$$

where γ is the amplitude attenuation coefficient, and I is the identity matrix. Because the loss term is proportional to the identity matrix, it commutes with the coupling

Hamiltonian. Over one complete Floquet period T, the Floquet operator is consequently

$$U_{NH}(T) = e^{-\gamma T} U(T). \tag{62}$$

Thus, each Floquet quasi-energy acquires the same imaginary component,

$$\epsilon_{NH} = \epsilon - i\gamma \tag{63}$$

The real parts of the quasi-energies and the corresponding Floquet eigenvectors remain unchanged, whereas all states acquire an identical loss-induced linewidth. Therefore, uniform isotropic material absorption does not selectively broaden or destabilize the corner states relative to the bulk states.

After N Floquet periods, the field amplitude evolves as

$$|\psi_{NH}(NT)\rangle = e^{-\gamma NT} U(T)^N |\psi(NT)\rangle, \tag{64}$$

and the total optical power decreases according to

$$\frac{P(NT)}{P_0} = e^{-2\gamma NT}. \tag{65}$$

Because the attenuation factor is common to all modal components, the normalized modal distribution and the normalized $j \to -j$ conversion fidelity remain unchanged in this uniform-loss model. Material absorption instead reduces the absolute converted signal and therefore limits the experimentally accessible propagation distance.

Using the experimentally characterized complex effective refractive index of the RG-resin waveguide at the operating frequency, the corresponding intensity attenuation is approximately 1.67 dB mm^{-1}. Taking a dynamic range of approximately 100 dB for the envisioned VNA-based measurement as an upper-bound detection criterion gives an ideal detectable propagation distance of approximately 59.8 mm. For the coupling parameters considered here, this distance corresponds to approximately two Floquet periods and is sufficient to resolve the characteristic $j \to -j$ reversal shown in Fig. 4a. In practice, additional insertion and coupling losses will further reduce the experimentally accessible distance. Lower-loss printable materials or optimized resin

formulations would therefore allow the Floquet evolution to be observed over a larger number of driving periods.

The corresponding complex quasi-energy spectrum and propagation-loss analysis are shown in Supplementary Fig. S10.

Supplementary Note 15 | Conversion efficiency between vortex modes of different orders.

We further consider the conversion efficiency between vortex modes of different orders in two waveguides with different radii. The relevant modal basis is formed by two spin–orbit-coupled vortex modes in each waveguide. After absorbing the fixed coupling phases into the modal basis, the coupling dynamics can be described by the corresponding coupled-mode Hamiltonian.

For excitation of a single input vortex mode, complete conversion to an individual target mode can in principle be achieved when the undesired coupling channels are suppressed and only one effective conversion channel remains. For an effective two-mode coupling process with coupling coefficient κ, the modal amplitudes evolve as

$$\begin{aligned} A_1(z) &= \cos^2(\kappa z), \\ A_2(z) &= -e^{i\,2(l+1)\theta_{12}} \sin^2(\kappa z)\,, \\ A_3(z) &= -e^{i(l-m)\theta_{12}} \sin(\kappa z)\cos(\kappa z), \\ A_4(z) &= -e^{i(l+m+2)\theta_{12}} \sin(\kappa z)\cos(\kappa z). \end{aligned} \tag{66}$$

giving

$$\begin{aligned} |A_1|^2 &= \cos^4(\kappa z), \\ |A_2|^2 &= \sin^4(\kappa z), \\ |A_3|^2 = |A_4|^2 &= \sin^2(\kappa z)\cos^2(\kappa z). \end{aligned} \tag{67}$$

The theoretical conversion efficiency therefore reaches unity at $z = \pi/(2\kappa)$.

In the general dual-waveguide configuration considered in this work, however, several symmetry-allowed coupling channels coexist simultaneously. The conversion dynamics must therefore be obtained from the complete coupled-mode equation

$$i\frac{d}{dz}A(z) = HA(z), \quad (68)$$

where $A(z)$ contains the modal amplitudes of all participating vortex modes. The conversion efficiency into a selected target mode mm is defined as $\eta_n = max|A_n(z)|^2$. The representative phase-matched dual-waveguide structure used in Fig. 5, the two relevant vortex-mode components in waveguide 2 reach maximum conversion efficiencies of approximately 63.7% and 72.9%, respectively. The values remain below unity because both target and non-target coupling channels are simultaneously present in this geometry. Thus, complete conversion is allowed in the ideal single-channel limit, whereas the conversion efficiency of a practical multi-channel coupler is determined by the relative strengths of the competing coupling coefficients. Besides, the conversion efficiency can be further increased by engineering the waveguide radii, inter-waveguide spacing, and modal overlap.

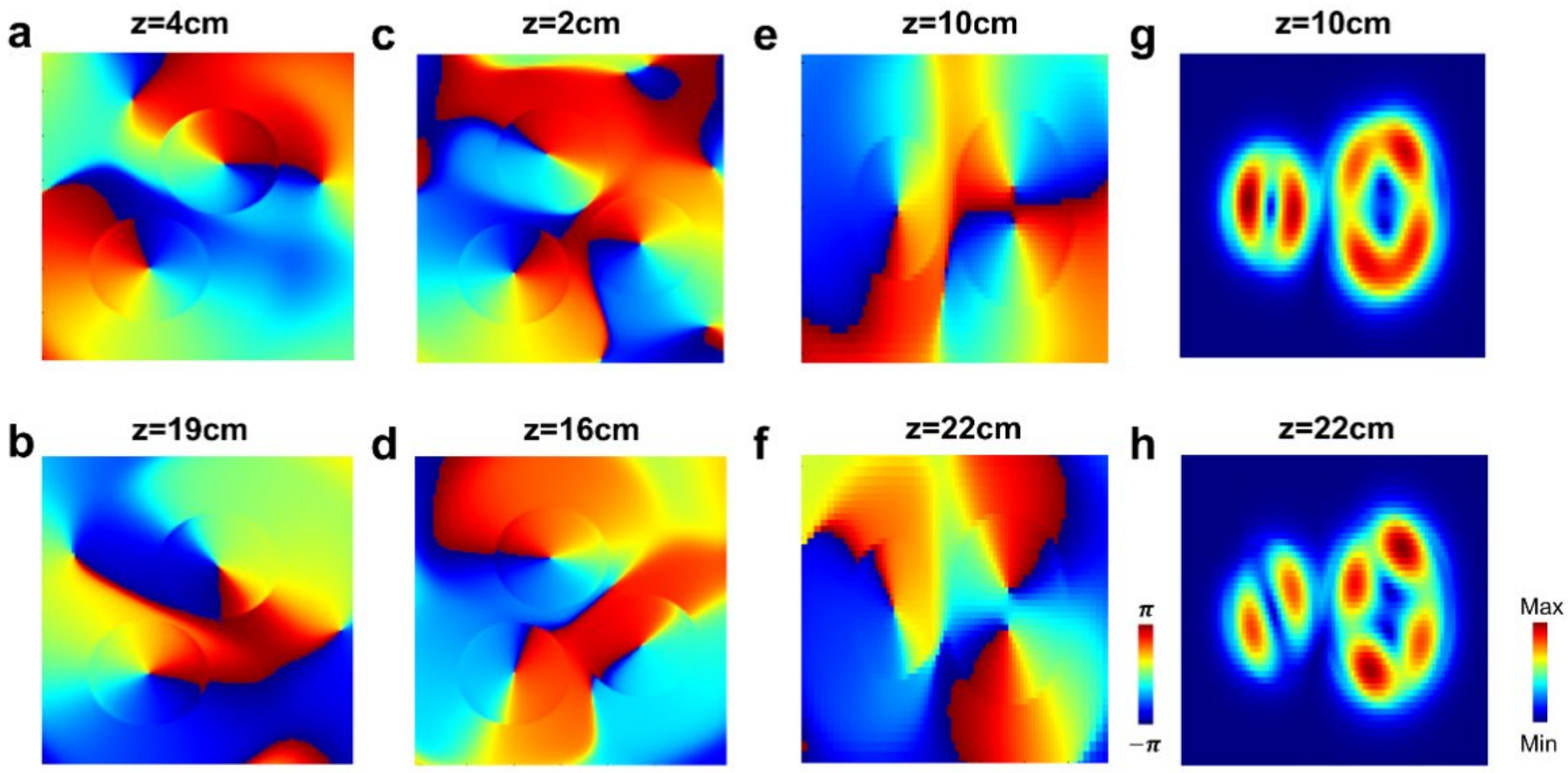

Fig S1. | Simulated electric field magnitude and phase distribution (x-polarized component)

a, b, Phase distribution at propagation distances at $z = 4cm$ and $z = 19cm$ for a two-waveguide structure with identical radii. **c, d**, Phase distribution at propagation distances at $z = 2cm$ and $z = 16cm$ for a three-waveguide structure with identical radii. **e, f**, Phase distribution at propagation distances at $z = 10cm$ and $z = 22cm$ for a two-waveguide structure with non-identical radii. **g, h**, Magnitude distribution at propagation distances at $z = 10cm$ and $z = 22cm$ for a two-waveguide structure with non-identical radii.

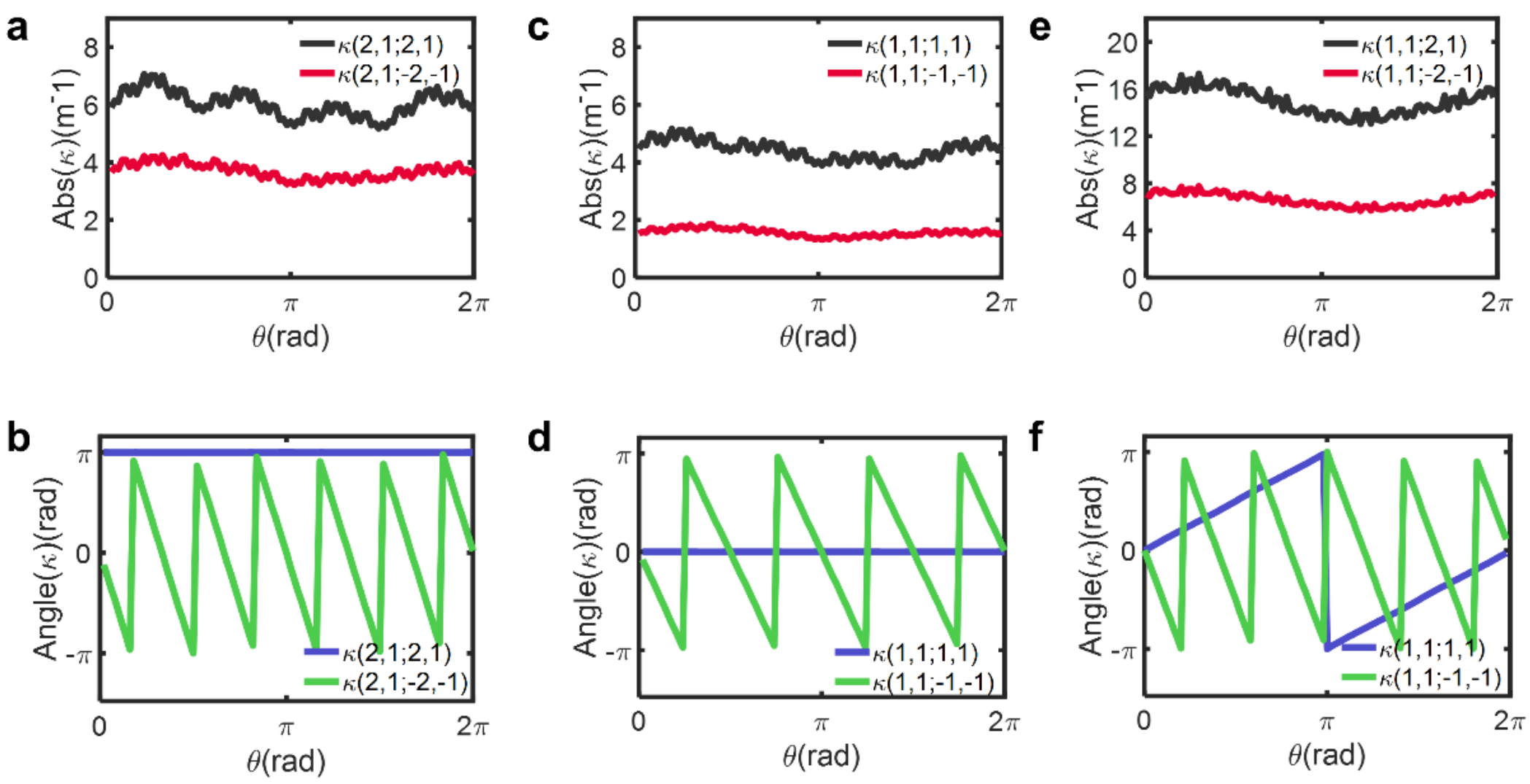

Fig S2. | The theoretical results for the magnitude and phase of the coupling coefficients.

a,**b**, Magnitude and phase of the coupling coefficients for a two-waveguide structure with identical radii, with incident light carrying OAM order $l = 2$. **c,d**, Magnitude and phase of the coupling coefficients for a two-waveguide structure with identical radii, with incident light carrying OAM order $l = 1$. **e,f,** Magnitude and phase of the coupling coefficients for a two-waveguide structure with non-identical radii, with incident light carrying OAM order $l = 1$.

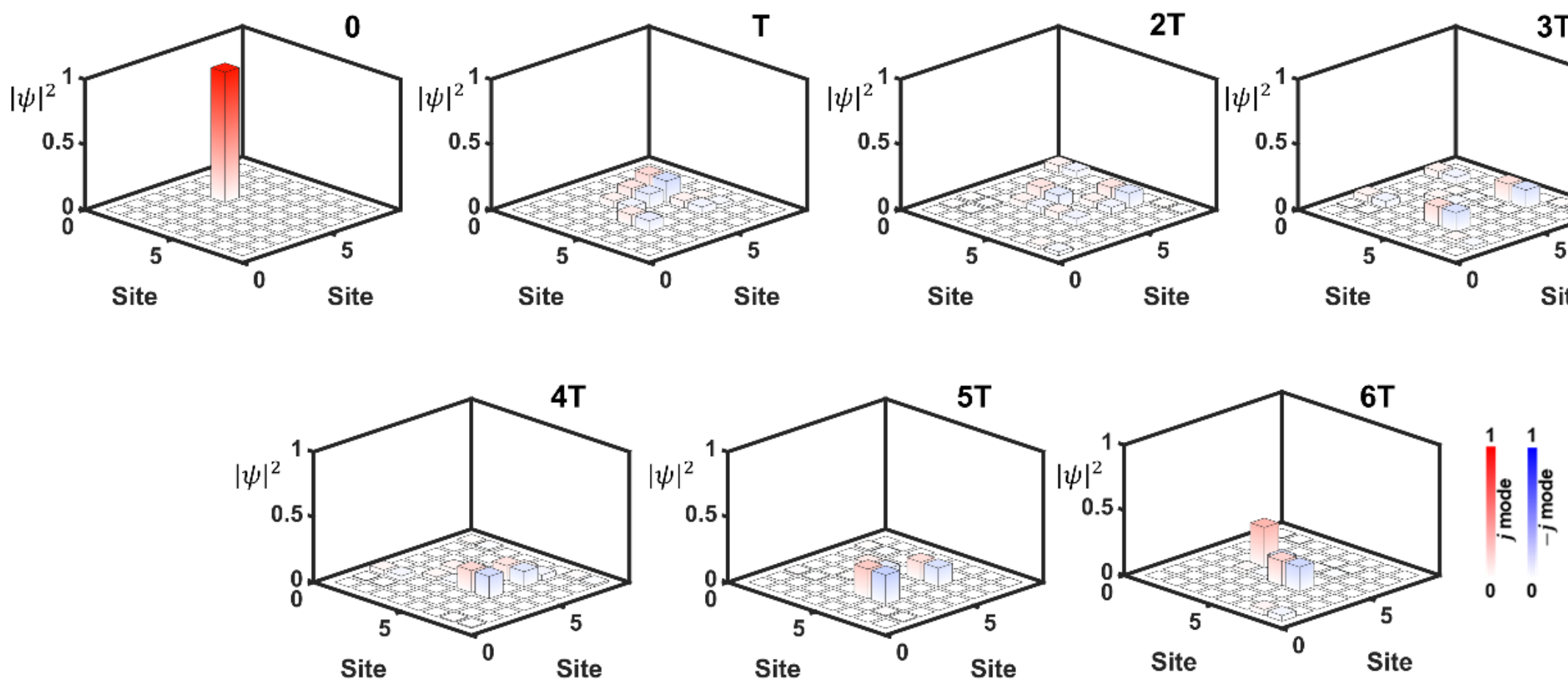

Fig S3. | Evolution of the bulk state under coupling parameters $c_1 = \frac{3\pi}{5}$ and $c_2 = \frac{\pi}{4}$.

When light is injected into a central waveguide, in contrast to the localized behavior of edge and corner states, the excitation spreads into the bulk of the array after several driving periods.

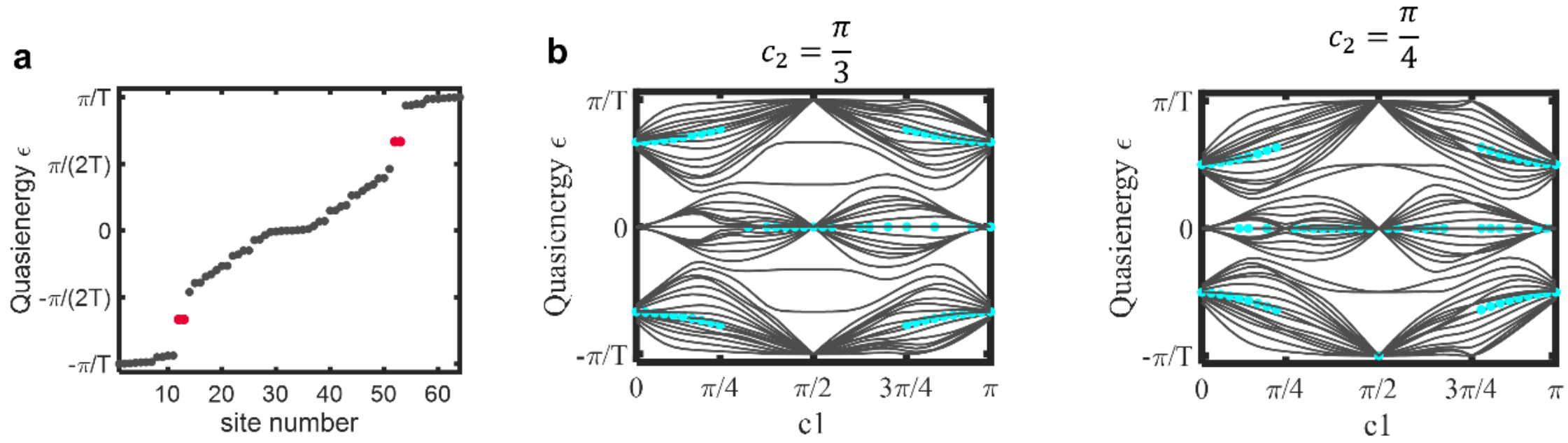

Fig S4. | Quasi-energy spectra under distinct coupling conditions. a, Quasi-energy spectrum of a finite structure with coupling parameters $c_1 = \frac{3\pi}{5}$ and $c_2 = \frac{\pi}{3}$, with random perturbations introduced into the system. **b**, Numerically calculated quasi-energy spectra of a finite structure of 32 waveguides under different coupling parameters, corresponding to a two-waveguide structure with non-identical radii.

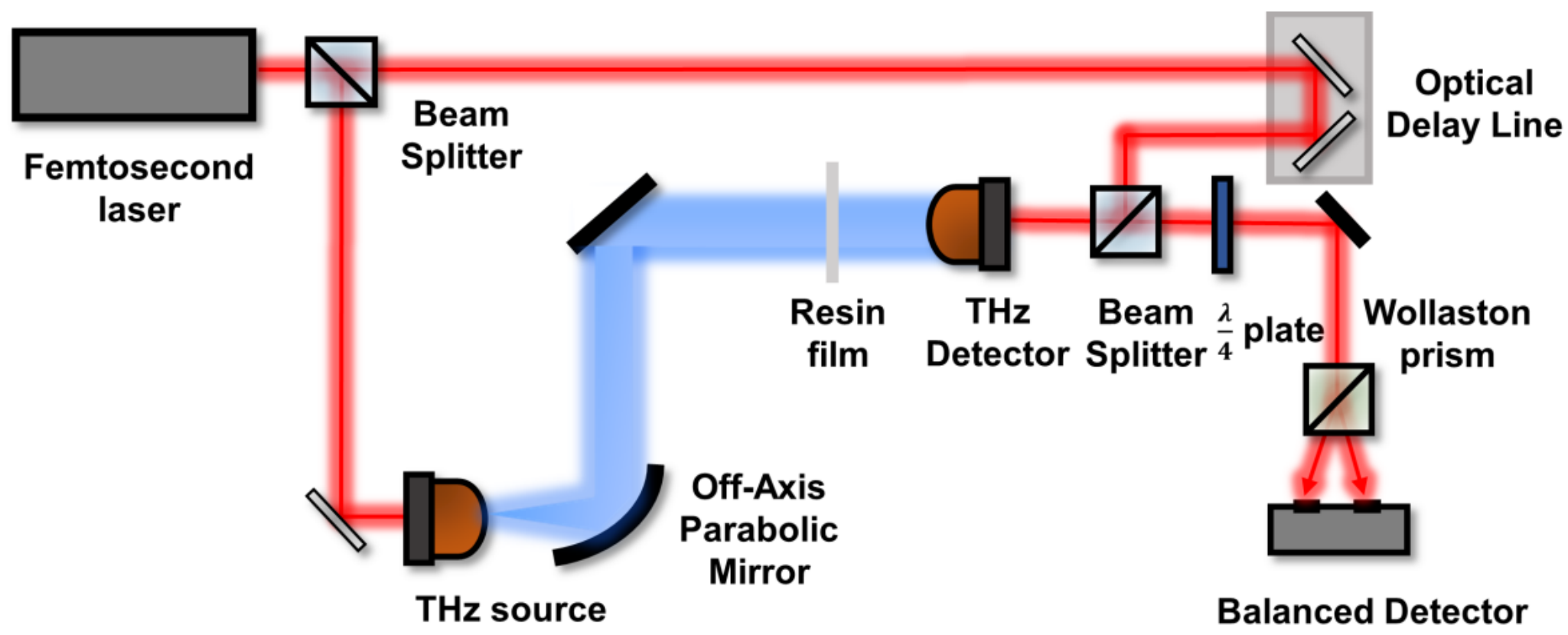

Fig S5. | Experimental setups of the THz-TDS systems characterization of RG resin films.

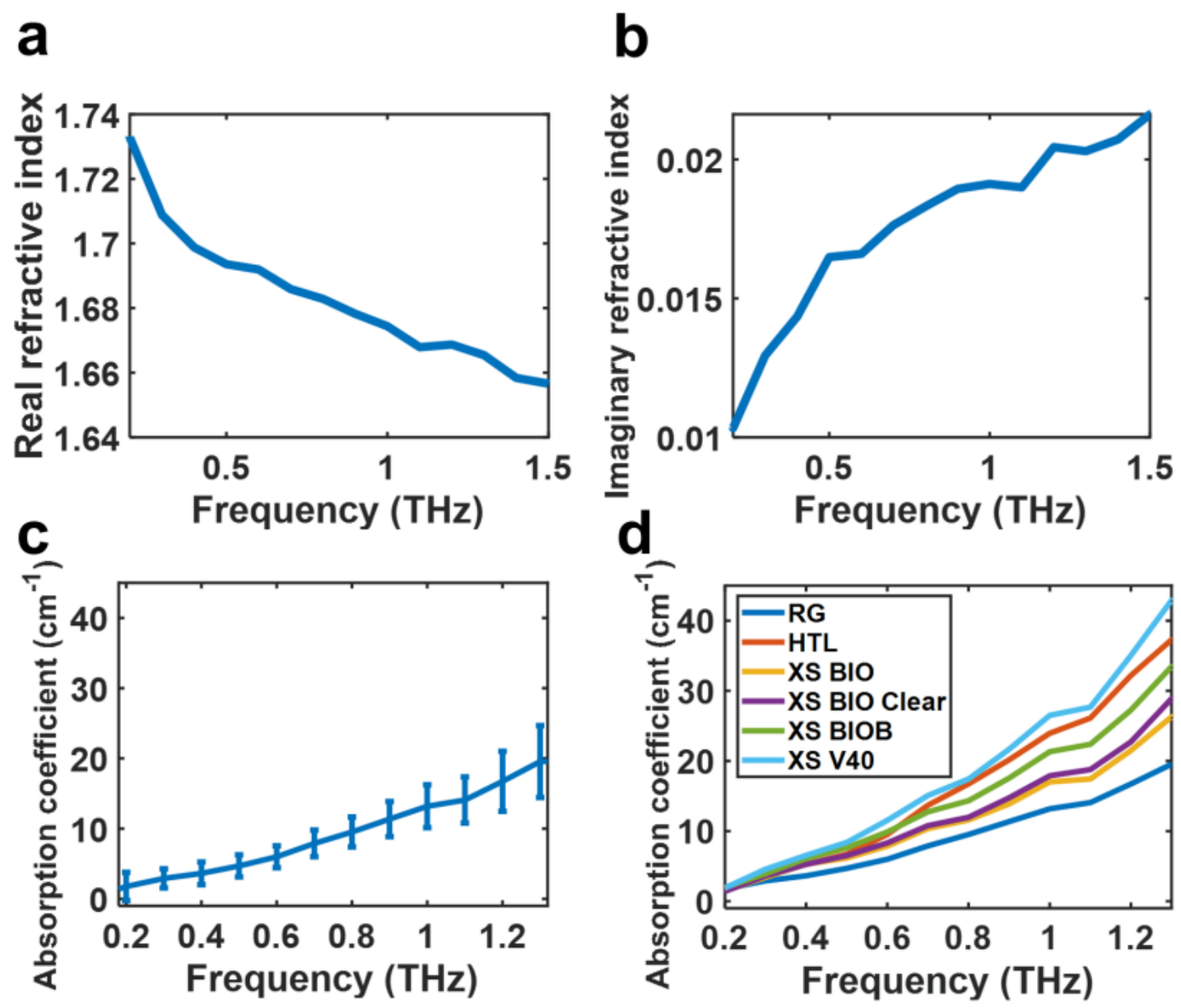

Fig S6. | Terahertz characterization of RG resin films. a, Real part of the refractive index of a 1-mm-thick RG resin film obtained from terahertz time-domain spectroscopy. **b**, Imaginary part of the refractive index of the same film. **c**, Absorption coefficient of the RG resin extracted from the THz-TDS measurements, showing values consistent with previously reported absorption coefficients of resin-based materials in the terahertz band[11].At 0.4 THz, an absorption coefficient of $\alpha = 3.66\ \mathrm{cm}^{-1}$ corresponds to an imaginary part of the refractive index of approximately n_i=0.022. **d**, Comparison of the absorption coefficients of several candidate resins measured by THz-TDS at different frequencies, showing that RG exhibits the lowest absorption among the tested materials and is therefore selected for sample fabrication.

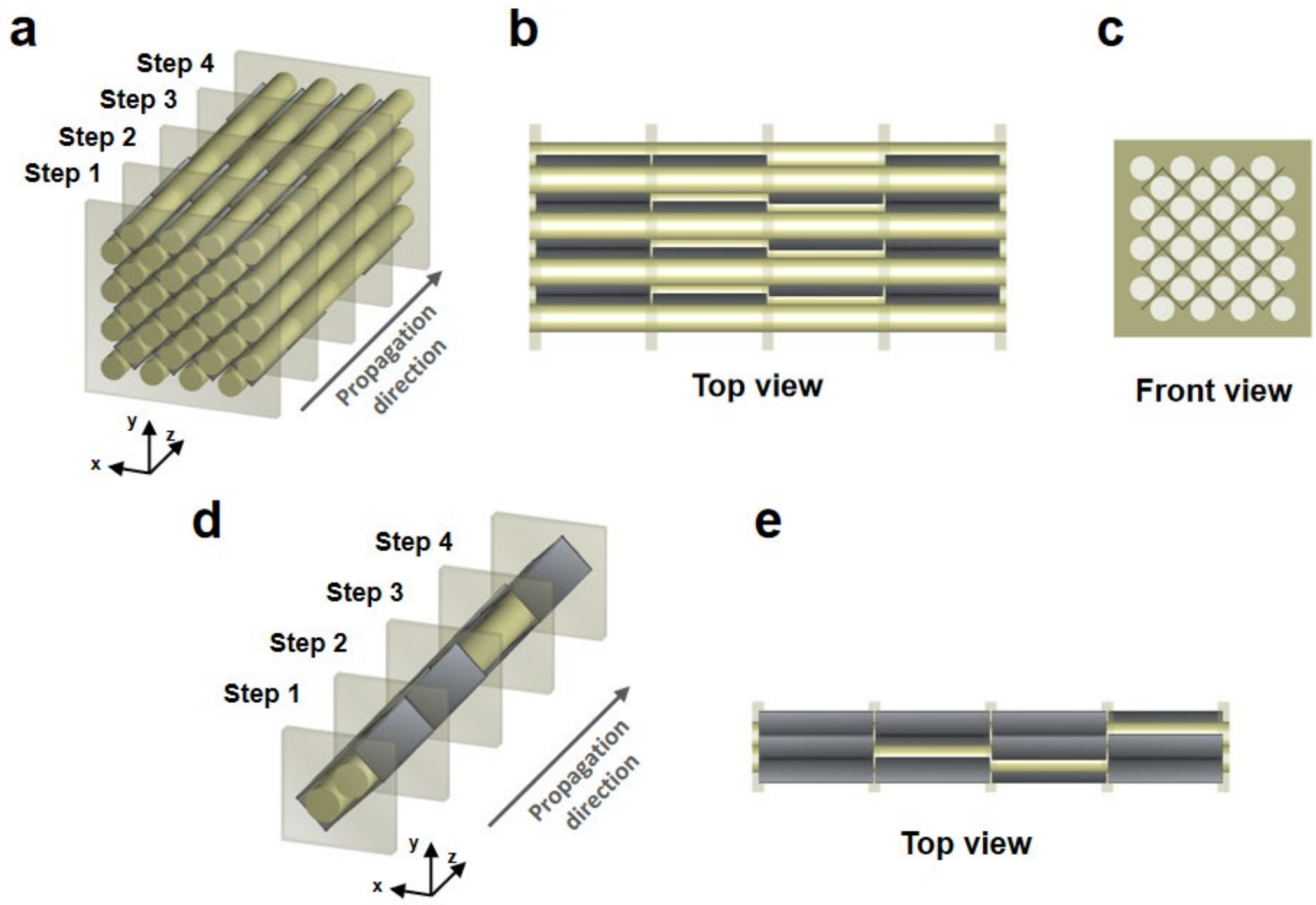

Fig. S7 | Structural implementation of the four-step Floquet lattice using dielectric waveguides and metallic coupling barriers. a, Three-dimensional schematic of the full waveguide array over one Floquet period, divided into four longitudinal steps. The dielectric waveguides are periodically supported by transverse resin plates, while metallic barriers are selectively inserted between neighboring waveguides to suppress the corresponding couplings in each step; waveguide pairs without a metallic barrier retain their nearest-neighbor coupling. **b,c,** Top and front views of the full structure, respectively, showing the longitudinal arrangement of the metallic barriers and the waveguides. **d,** Representative two-waveguide local unit illustrating the same four-step implementation, in which the presence or absence of the metallic barrier switches the corresponding inter-waveguide coupling off or on. **e,** Top view of the local unit, showing the step-dependent longitudinal distribution of the metallic barriers.

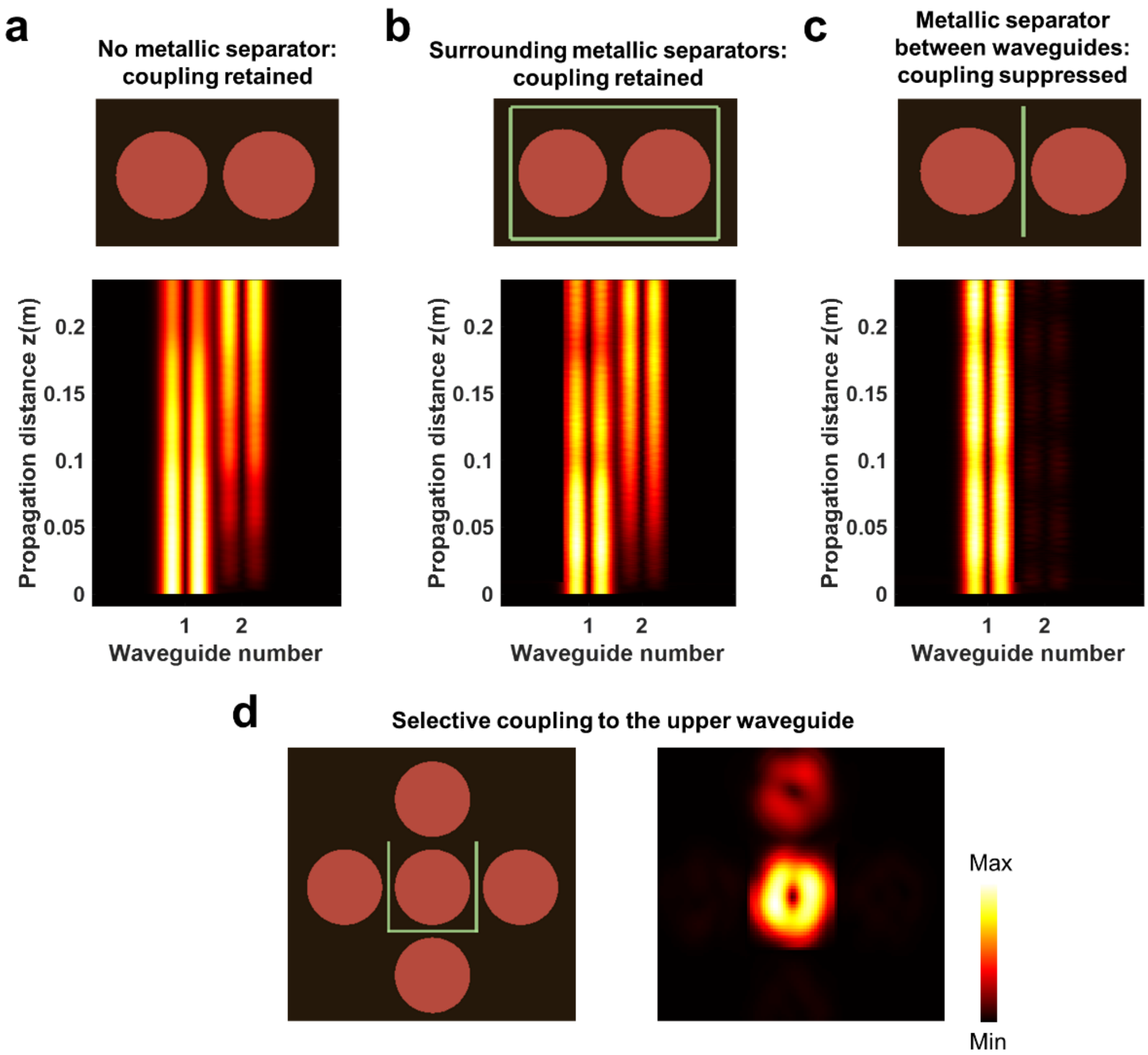

Fig. S8 | Selective control of inter-waveguide coupling using metallic separators. a, Two adjacent waveguides without metallic separators, showing the normal coupling between the waveguides. **b,** Two adjacent waveguides with metallic separators placed around the pair, while no separator is introduced between them. The coupling between the two waveguides remains nearly unchanged, indicating that the surrounding separators have only a weak influence on the intended coupling channel. **c,** A metallic separator placed directly between the two waveguides suppresses the evanescent-field overlap and effectively inhibits their coupling. **d,** Selective coupling in a five-waveguide configuration consisting of a central waveguide and its four nearest neighbors. Metallic separators are introduced along the left, right, and lower coupling channels, thereby suppressing these three channels, while the unblocked upper channel remains coupled, as confirmed by the simulated field distribution.

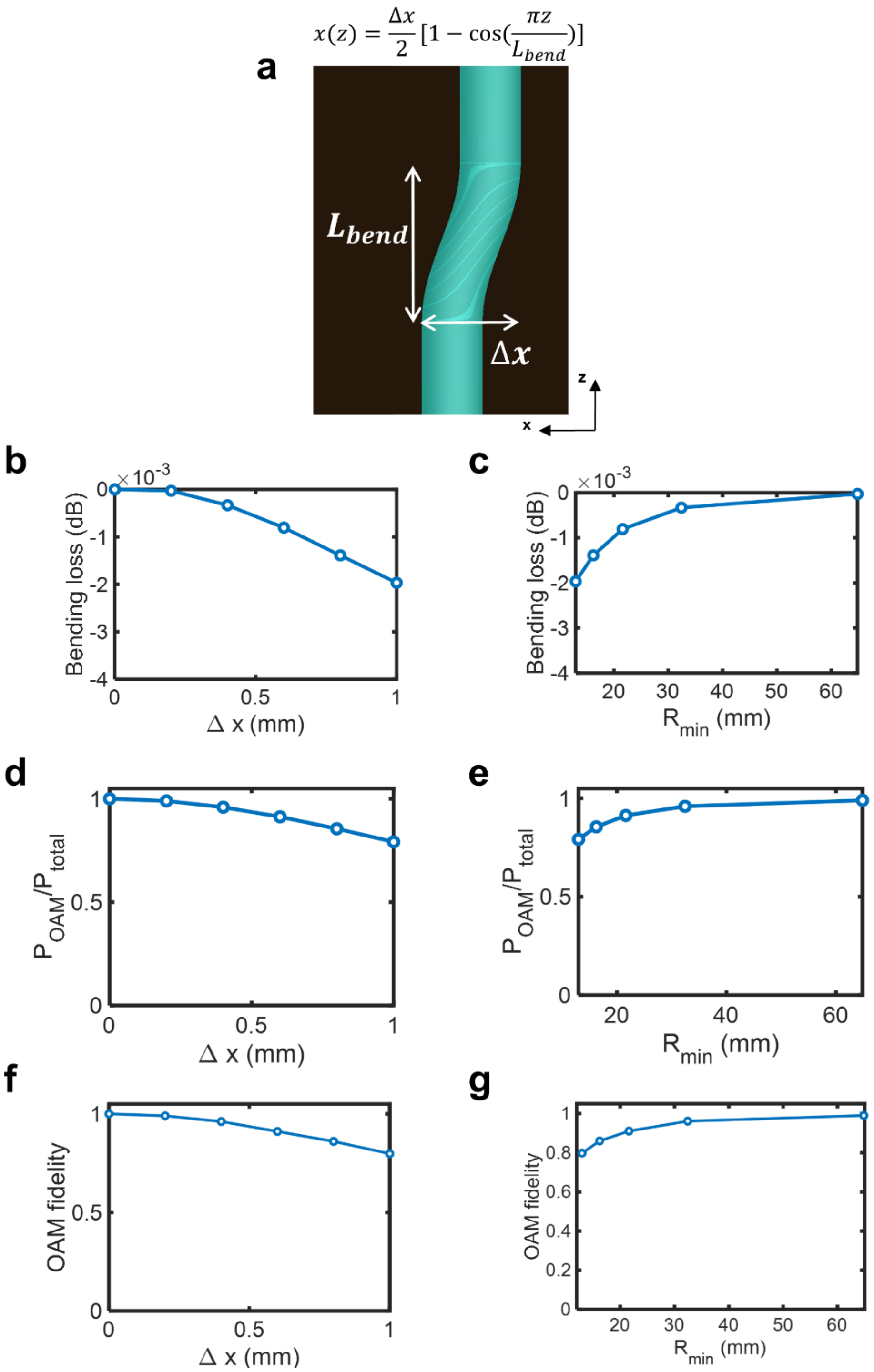

Fig. S9 | Quantitative evaluation of bending-induced loss and mode distortion. a, Schematic of the bending trajectory used in the full-wave FDTD simulations. The lateral displacement is described by $x(z) = \frac{\Delta x}{2}\left[1 - cos\left(\frac{\pi z}{L_{bend}}\right)\right]$, where Δx is the total lateral displacement and L_{bend} is the longitudinal length of the bending section. **b, c,** Bending loss as a function of the lateral displacement Δx and the corresponding minimum bending radius R_{min} espectively. **d, e,** Fraction of the transmitted power

remaining in the target OAM-mode subspace, P_{OAM}/P_{total}, as a function of Δx and R_{min}. **f, g,** Corresponding OAM-mode fidelity as a function of Δx and R_{min}. For the maximum displacement required by the four-step protocol, $\Delta x = 0.4$ mm, the bending loss remains below 10^{-3} dB, while more than approximately 95% of the transmitted power remains in the target OAM-mode subspace with a high OAM fidelity, indicating that the required bending introduces only weak radiative loss and mode distortion.

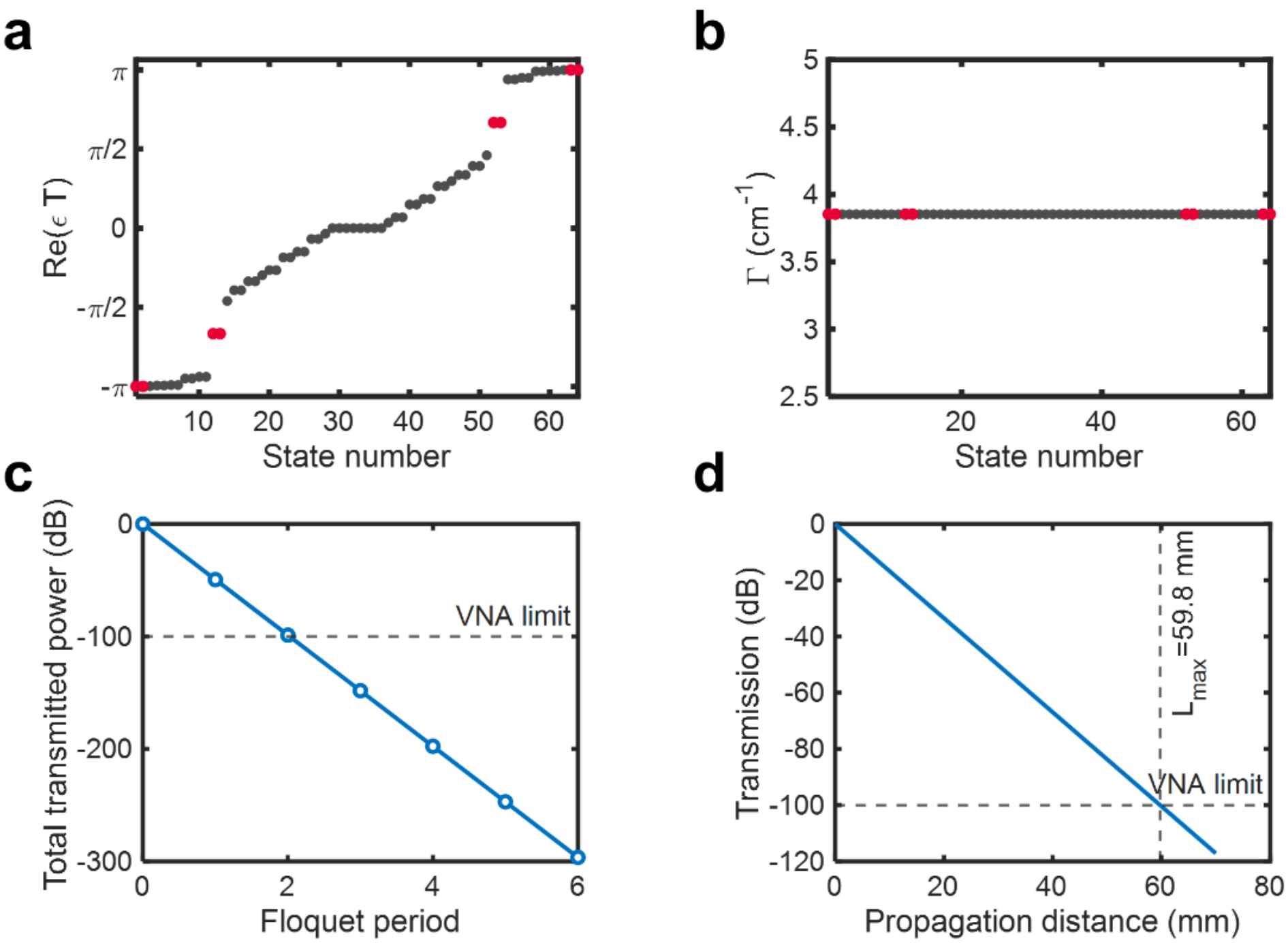

Fig. S10 | Loss-induced Floquet quasi-energy linewidth and experimentally accessible propagation range. a, Real part of the Floquet quasi-energy spectrum, Re(ϵT), with the corner states highlighted in red. **b,** Corresponding loss-induced quasi-energy linewidth Γ. Because the material loss is introduced as a spatially uniform attenuation term, all Floquet eigenstates acquire the same linewidth. **c,** Total transmitted power as a function of the number of Floquet periods, with the dashed line indicating the 100-dB dynamic-range limit of the VNA. **d,** Transmission versus physical propagation distance. The estimated maximum detectable propagation length is approximately 59.8 mm, corresponding to about two Floquet periods for the present coupling strength.

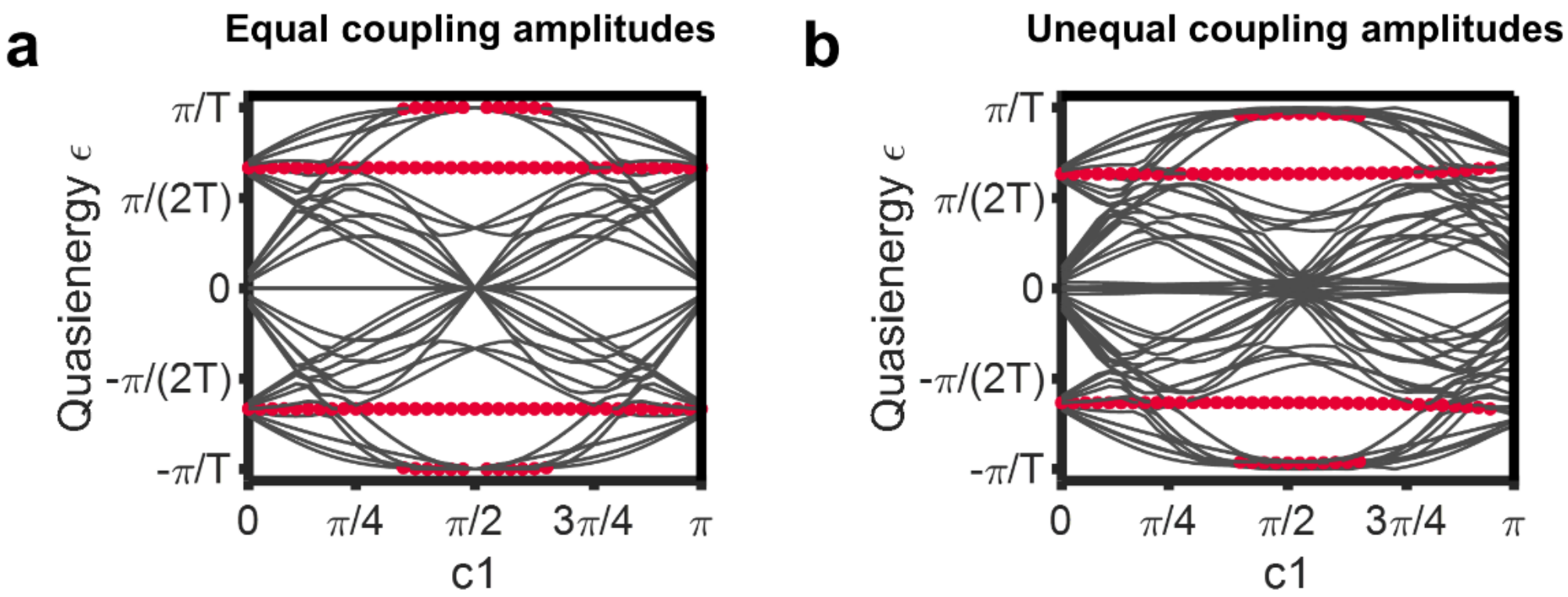

Fig. S11 | Quasi-energy spectra for different relative coupling amplitudes between OAM modes. a, Quasi-energy spectrum at fixed $c_2 = \pi/3$ when the coupling amplitudes between the same-sign and opposite-sign OAM modes are equal. **b,** Corresponding spectrum when these two coupling amplitudes are unequal. The red markers indicate the corner states. In both cases, the corner states persist, although their quasi-energies shift when the relative coupling amplitudes are changed. Therefore, the Floquet OAM conversion remains supported, while the ratio between c_1 and c_2 required for a specific multiple-period oscillation needs to be reidentified according to the actual ratio of the same-sign and opposite-sign OAM coupling amplitudes.

a

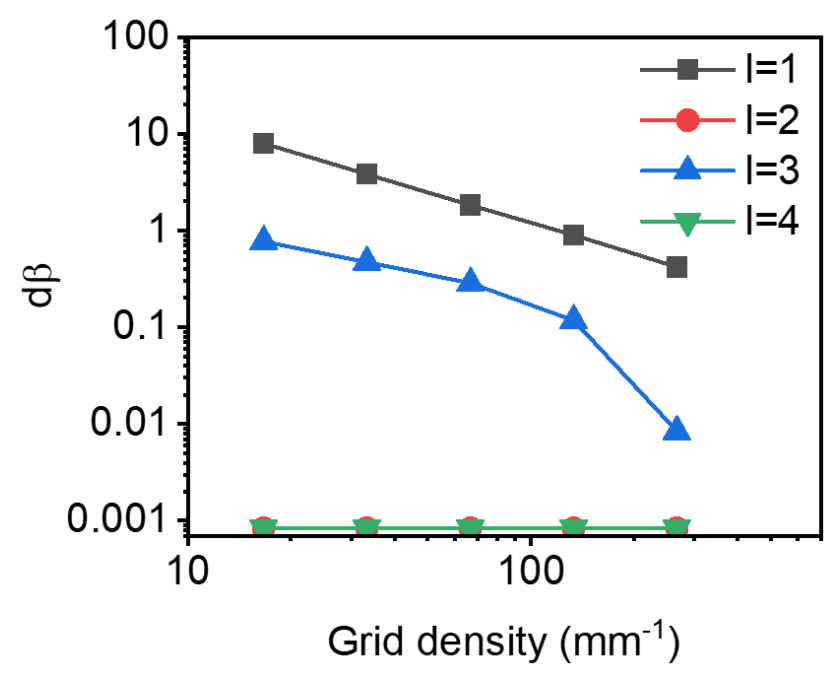

Fig. S12 | Dependence of the propagation-constant mismatch on the FDE simulation resolution. a, Calculated $d\beta$ as a function of the grid density for OAM modes with topological charges $l = 1–4$. The mismatch decreases with increasing grid density for the odd-order modes ($l = 1$ and $l = 3$), whereas that of the even-order modes ($l = 2$ and $l = 4$) remains close to the numerical precision limit of the FDE solver.

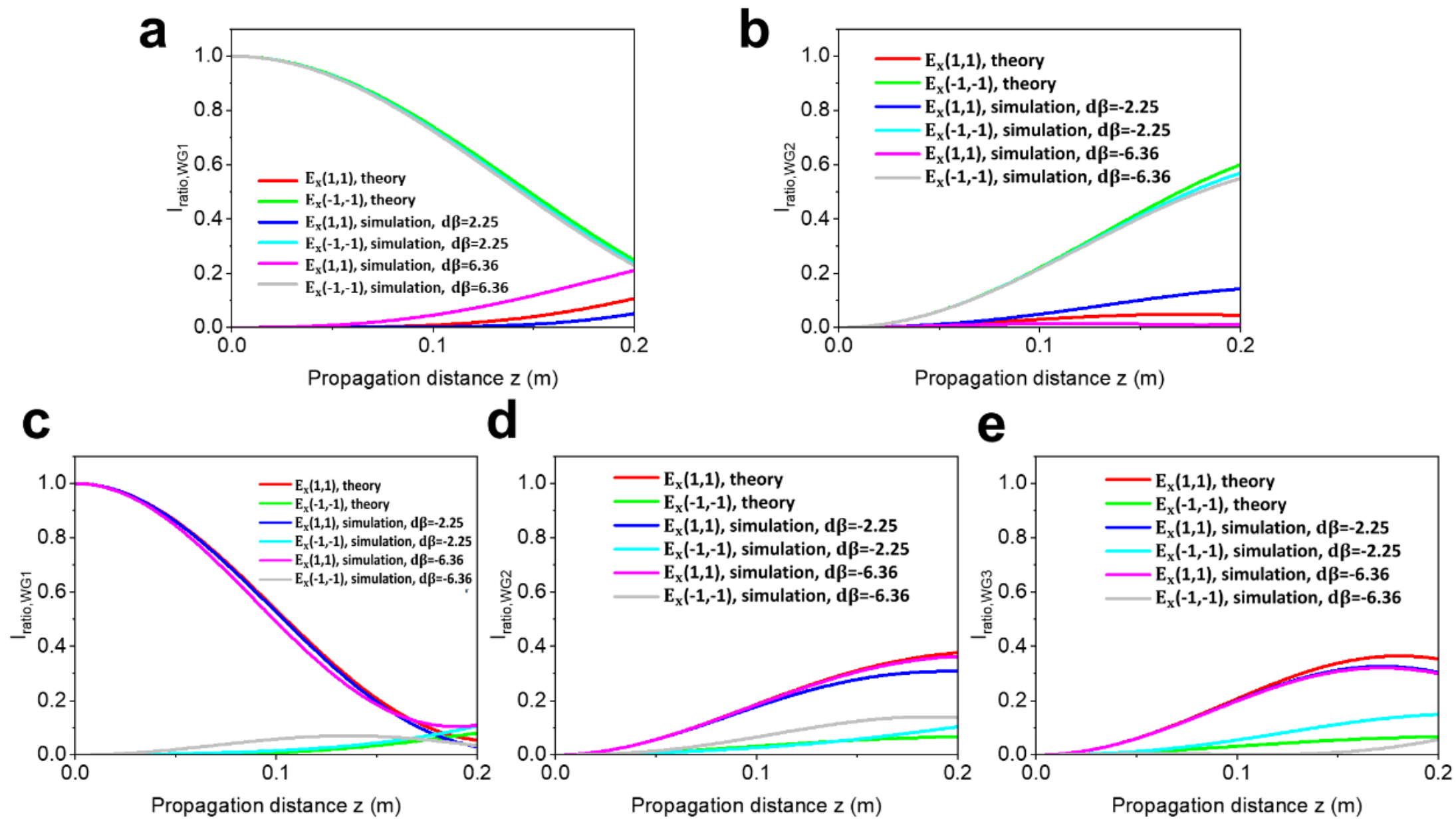

Fig. S13 | Influence of the propagation-constant mismatch on the coupling dynamics. a, b, Evolution of the modal intensity fractions in waveguides 1 and 2, respectively, for the dual-waveguide system. **c–e,** Evolution of the modal intensity fractions in waveguides 1–3, respectively, for the three-waveguide system. The theoretical results correspond to the ideal case of $d\beta = 0$, while the simulated results are calculated for finite propagation-constant mismatches of $d\beta = \pm 2.25$ and ±6.36, illustrating the deviation of the coupling dynamics from the ideal case as $|d\beta|$ increases.

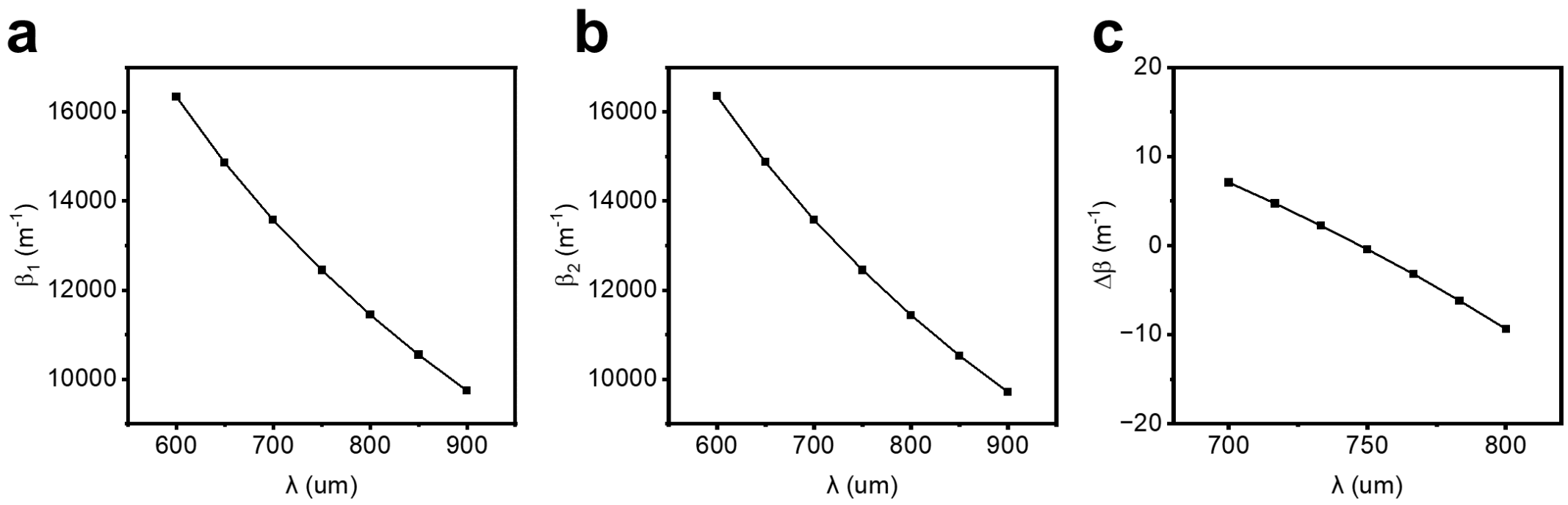

Fig. S14 | Waveguide dispersion and wavelength-dependent propagation-constant mismatch for the cross-OAM coupling scheme. a, b, Calculated propagation constants β_1 and β_2 of the phase-matched modes in waveguides 1 and 2, respectively, as functions of wavelength, obtained using the FDE eigenmode solver. **c,** Corresponding propagation-constant mismatch, $\Delta\beta = \beta_1 - \beta_2$, around the designed phase-matching wavelength. The two modes satisfy $\Delta\beta \approx 0$ near $\lambda = 750$ µm, while wavelength detuning produces a finite phase mismatch because of their different waveguide dispersions.

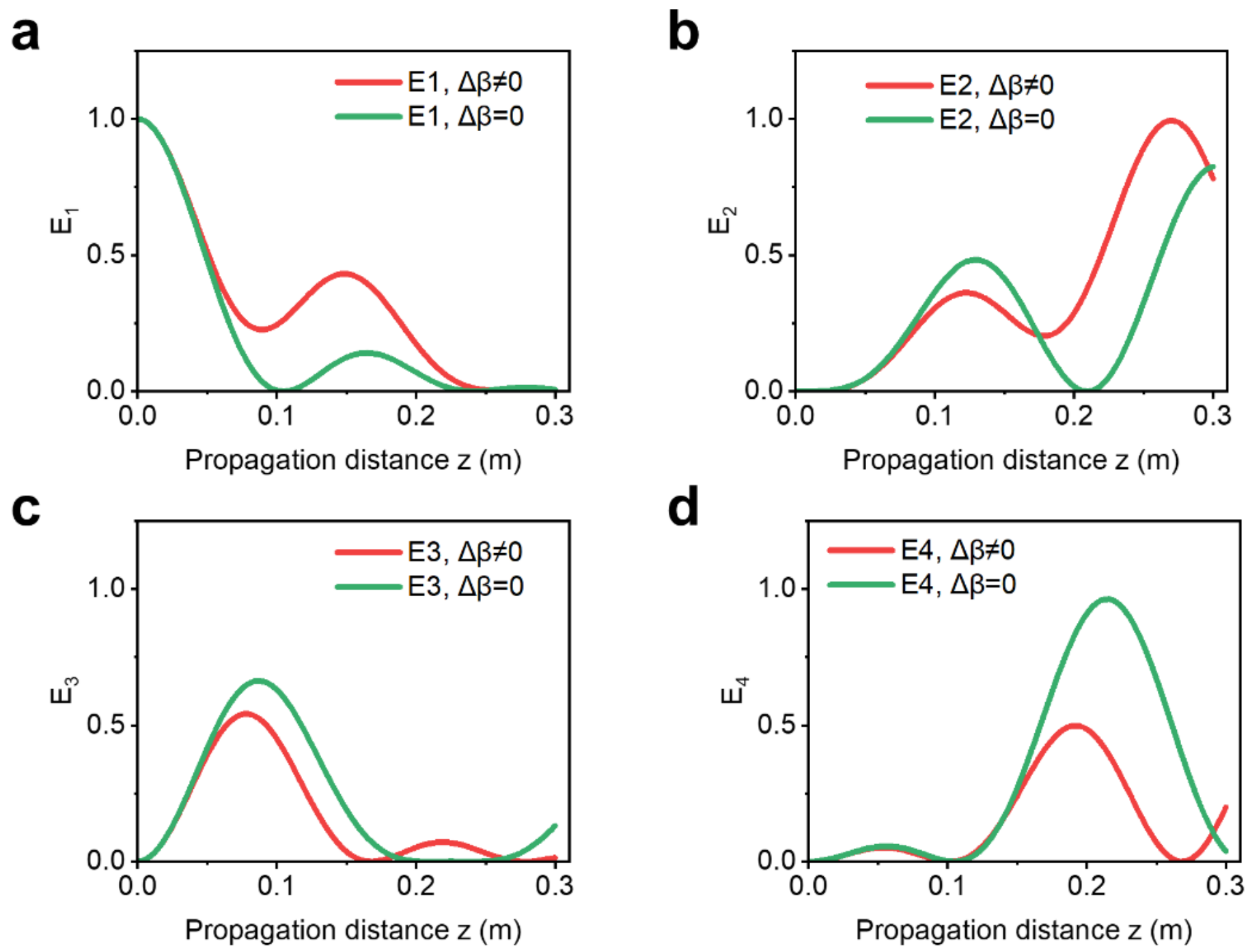

Fig. S15 | Influence of propagation-constant mismatch on the cross-OAM coupling dynamics. a–d, Evolution of the modal intensities $E_1 - E_4$ respectively, as functions of propagation distance for the ideal phase-matched case $\Delta\beta = 0$ and the case including a finite propagation-constant mismatch $\Delta\beta \neq 0$. The mismatch modifies both the conversion amplitudes and the positions of the conversion maxima. In particular, the maximum conversion of the E_4 mode is substantially reduced by $\Delta\beta$, and is therefore used to quantify the operational bandwidth of the cross-coupling resonance.

Table S1 | $j \to -j$ conversion efficiencies at representative propagation distances. The local conversion efficiency is evaluated at the initially excited site as the fraction of the local power occupying the $-j$ mode, whereas the global conversion efficiency is evaluated as the fraction of the total lattice power occupying all $-j$ modes. Results are shown for $c_1 = \frac{3\pi}{5}, c_2 = \frac{\pi}{4}$ and $c_1 = \frac{3\pi}{5}, c_2 = \frac{\pi}{3}$.

$c_1 = 3\pi/5$ and $c_2 = \pi/4$				
Propagation distance	2T	6T	10T	14T
$j \to -$ conversion efficiency at the initially excited site	99.884	99.720	99.962	99.701
Global $j \to -j$ conversion efficiency	96.701	94.965	98.093	94.812
$c_1 = 3\pi/5$ and $c_2 = \pi/3$				
Propagation distance	3T	9T	15T	21T
$j \to -j$ conversion efficiency at the initially excited site	99.696	99.440	99.850	99.214
Global $j \to -j$ conversion efficiency	94.769	93.021	96.267	91.826
